# Clinical burden of propionic acidemia in the United States: a claims-based study by age stratum

**DOI:** 10.1186/s13023-025-03901-2

**Published:** 2025-07-15

**Authors:** Geetanjoli Banerjee, Sue Perera, Fan Mu, Erin Cook, Mu Cheng, Adina Zhang, Jessie Jie Lan, Lin Zou, Vanja Sikirica

**Affiliations:** 1 Moderna Therapeutics, Inc., 325 Binney St, Cambridge, Massachusetts 02142 USA; 2 Analysis Group, Inc., 111 Huntington Avenue, 14th floor, Boston, Massachusetts 02199-7668 USA; 3 5 Vaughn Dr Princeton, NJ 08540 for Moderna Therapeutics, Inc., NJ, USA

**Keywords:** Propionic acidemia, Metabolic decompensation event, Propionyl coenzyme A carboxylase, Clinical outcomes, Age stratification, Administrative claims data

## Abstract

**Background:**

Patients with propionic acidemia (PA) may face recurrent metabolic decompensation events (MDEs) and multisystemic complications. This study compared characteristics and clinical outcomes of patients with PA and matched non-PA controls by age stratum.

**Methods:**

Patients with PA from the US IQVIA PharMetrics Plus claims database (10/2015‒6/2022) had their follow-up time partitioned into age strata (0‒2, 3‒6, 7‒12, 13‒17, ≥ 18 years) and were matched 1:1 to randomly selected non-PA controls within each stratum. MDEs were identified as hospitalizations with claims for hyperammonemia and/or metabolic acidosis. Hospitalizations with claims for PA signs and symptoms were evaluated.

**Results:**

Among 191 patients with PA and 230 matched non-PA controls (median follow-up: 2.7 years), patients with PA had more comorbidities (neurologic/nervous system, cytopenias, growth, metabolism, cardiac system; listed in order of frequency) across all age strata. The overall MDE rate for patients with PA was 0.5 per patient-year (PPY) while hospitalizations with various PA signs and symptoms ranged from 0.3 to 0.6 PPY. MDE rates were highest in those aged 3‒6 years (1.4 PPY), lowest in the 13‒17 years stratum (0.1 PPY), and rose again in adults (0.2 PPY). Patients with MDEs (31.4%) had a significantly higher burden of PA-related symptoms and comorbidities than those without; both groups showed even greater differences when compared to controls.

**Conclusions:**

Patients with PA across all age strata, with and without MDEs, experience a substantial burden of disease-related comorbidities, complications, and healthcare visits compared with matched non-PA controls, which highlights the need for improved clinical outcomes in these patients.

**Supplementary Information:**

The online version contains supplementary material available at 10.1186/s13023-025-03901-2.

## Background

Propionic acidemia (PA) is a rare inherited metabolic disorder (diagnosed in < 1/100,000 newborns in the United States [US] [1]) caused by pathogenic mutations in the *PCCA* and *PCCB* genes, which encode the two subunits of the mitochondrial enzyme propionyl coenzyme A (CoA) carboxylase (PCC) [2]. PCC deficiency leads to reduced catabolism of branched-chain amino acids, odd-chain fatty acids, and cholesterol, with consequent accumulation of toxic metabolites such as propionyl-CoA, ammonia, and organic acids in tissue and body fluids, as well as depletion of tricarboxylic acid (TCA) cycle intermediates and substrates [3]. This can result in metabolic crises or decompensation– broadly defined as biochemical instability (e.g., acidosis with high anion gap, ketonuria, hypoglycemia, and hyperammonemia)– which can be life-threatening and may lead to eventual organ failure [4]. 

Multiple organ systems are affected in PA and the clinical presentation is heterogeneous, often differing based on the timing of disease onset [2]. In cases with neonatal onset, infants may exhibit poor feeding, lethargy, cytopenia, hypotonia, and failure to thrive, which can progress to encephalopathy, seizures, and coma [5, 6]. The less common later-onset form of PA manifesting in older infants, children, and adults tends to have a milder phenotype that includes growth and motor impairment, protein intolerance, vomiting, hematologic complications, intellectual disability, pancreatitis, renal failure, and cardiomyopathy [6]. Acute metabolic decompensation events (MDEs) induced by catabolic stressors such as illness, infection, injury, surgery, or excessive protein intake can occur in patients of any age and may be fatal without timely and appropriate intervention [7, 8]. 

Current management approaches for PA focus on alleviating disease symptoms by targeting the effects of PCC deficiency [9, 10]. Hence, the standard of care for PA is medical nutrition therapy involving dietary modifications such as protein restriction or supplementation with vitamins, and supplemental therapy including the PCC cofactor biotin, or TCA cycle intermediates or substrate (L-carnitine) which promote protein breakdown [11, 12]. Carglumic acid is approved for the management of hyperammonemia; [13–16] antibiotics may be administered to suppress propionic acid-producing intestinal bacteria [17]. Liver transplantation can improve metabolic control in patients who experience frequent MDEs despite standard medical management, but it is not curative as it does not prevent dysfunction in other organs; [18–21] moreover, it is associated with non-trivial rates of complications and mortality [22]. Therefore, new therapeutic strategies that address the underlying cause of PA—i.e., the defective PCC enzyme—are needed to prevent the occurrence of MDEs and other complications, and thereby reduce morbidity and risk of mortality in patients. In this regard, experimental treatments involving enzyme replacement through gene [23, 24] or mRNA [25] delivery have been explored in preclinical studies [26], with a dual mRNA therapy currently in early-phase clinical trials [27, 28]. 

As a rare disease, there is limited information on the burden of PA from large studies in real-world settings. Most studies to date have been conducted in small samples, using historic data, or outside the US [1, 29–35]. To obtain more detailed insight into the disease burden in a large contemporary cohort, the present study compared the characteristics and clinical outcomes of patients with PA and matched non-PA control subjects in the US across age strata.

## Methods

### Data source

The data source for this study was the IQVIA PharMetrics Plus Claims database (October 1, 2015‒June 20, 2022), containing data on over 190 million unique beneficiaries including over 150 million who are covered by both medical and pharmacy plans. Diverse geographic areas, employers, providers, therapeutic areas, and payers across the US are represented in the database. The data are deidentified and comply with the patient confidentiality requirements of the Health Insurance Portability and Accountability Act (HIPAA).

### Study design

**Fig. 1:**
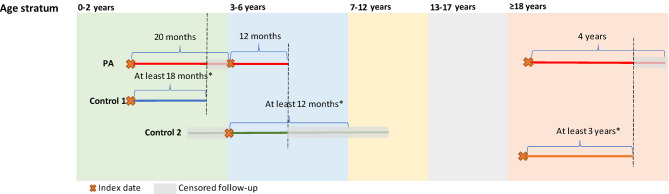
Study design and matching of patients with PA and non-PA control subjects. *The minimum follow-up for a control subject was the follow-up time of the matched patient with PA rounded down to the nearest 6-month increment, up to a maximum of 3 years. For example, for patients with PA with 12, 25, and 35 months of follow-up, matched control subjects were required to have at least 12 (2 × 6), 24 (4 × 6), and 30 (5 × 6) months of follow-up, respectively. Abbreviation: PA, propionic acidemia

This was a retrospective, observational cohort study evaluating clinical outcomes of patients with PA and a matched cohort of randomly selected control subjects without PA. Patients with PA were followed from their first observed PA diagnosis with their follow-up time partitioned into 0‒2, 3‒6, 7‒12, 13‒17, and ≥ 18 years age strata. A randomly selected control subject without PA was matched to a patient with PA within each age stratum. The observation period was defined for each stratum as follows (Fig. 1): the index date for the first age stratum was the date of the first observed PA diagnosis (PA cohort) or first medical or pharmacy claim (non-PA cohort); for all other age strata, the index date was the first day in the age stratum or start of a continuous enrollment period, whichever was earlier. Within each age stratum, the observation period spanned from the index date to the end of health plan enrollment, age stratum, or data availability, whichever was earliest.

### Study population

Inclusion criteria for the study were as follows: (1) at least one medical claim with a diagnosis code for PA (International Classification of Diseases, Tenth Revision, Clinical Modification [ICD-10-CM] code E71.121; PA cohort) or no diagnosis of PA (non-PA cohort) at any time on or after October 1, 2015; and (2) at least 6 months of continuous enrollment within any age stratum. Patients with missing age data were excluded.

Within each age stratum, control subjects were exactly matched 1:1 to patients with PA on age at index, index year ± 1 year (or ± 2 years when there was no match using 1 year), month of index date ± 1 month (to match on flu season—i.e., September to March), sex, geographic region (as determined by the 3-digit zip code, or state when there was no match using the zip code), and insurance plan type. Additionally, the two cohorts were partly matched on follow-up time, whereby the minimum follow-up for a control subject was the follow-up time of the matched patient with PA rounded down to the nearest 6-month increment, up to a maximum of 3 years. For example, for patients with PA with 12, 25, and 35 months of follow-up, matched control subjects were required to have at least 12 (2 × 6), 24 (4 × 6), and 30 (5 × 6) months of follow-up, respectively.

### Study measures

Patient demographic and clinical characteristics, including PA symptoms (i.e., anorexia/failure to feed, vomiting, metabolic acidosis, seizures, hyperammonemia), PA-related comorbidities and complications (i.e., metabolism-related conditions, cytopenias, growth complications, cardiac system conditions, neurologic and nervous system conditions), and other general comorbidities identified by ICD-10-CM and/or procedures codes were described during the 6-month period of continuous enrollment after the index date. Treatments received during the same period were identified by Generic Product Identifier (GPI)/National Drug Code (NDC) and/or procedure codes.

MDEs and hospitalizations with PA signs and symptoms for patients with PA were evaluated during the observation period. MDEs were identified using ICD-10-CM diagnosis codes for hyperammonemia and/or metabolic acidosis in an inpatient setting. Hospitalizations with PA signs and symptoms were identified based on ICD-10-CM codes for vomiting, anorexia/failure to feed, and/or seizures in an inpatient setting. A sensitivity analysis was conducted to evaluate MDEs and hospitalizations with PA signs and symptoms occurring not only in the inpatient setting but also in emergency room (> 24 h) setting. Liver transplantation was identified using diagnosis codes, procedure codes, or a treatment proxy of at least 6 months of continuous immunosuppressive therapy use (identified by GPI/NDC and/or procedure codes) without a gap > 60 days and evaluated at any time.

### Statistical analysis

Demographic characteristics, clinical characteristics, and treatments of patients with PA and matched non-PA control subjects were described using means and standard deviations (SDs) for continuous variables and frequencies and proportions for categorical variables within each age stratum during the 6-month post-index period. Differences in characteristics between the two cohorts were evaluated using the Wilcoxon signed-rank test (continuous variables) or chi-squared test (categorical variables). Liver transplantation at any time in patients with PA was reported using frequency and proportion; the earliest age at first evidence of liver transplantation (defined by procedure codes only) was described using mean and SD.

Rates of MDEs and hospitalization with PA signs and symptoms were calculated in patients with PA as the total number of events divided by total person-years of observation and are reported per patient-year (PPY) with 95% confidence intervals (CIs), overall and in each age stratum. Average length of hospital stays for MDEs and PA signs and symptoms were calculated among patients who experienced these events. It was assumed that non-PA subjects did not experience MDEs or PA signs and symptoms, so rates were not calculated for this cohort.

Analyses were stratified by the presence or absence of any MDE at any time among patients with PA. In addition, MDE rates before and after the start of the coronavirus disease 2019 (COVID-19) pandemic (i.e., March 2020) were described for patients with PA.

All statistical analyses were performed using SAS Enterprise 7.1 software (SAS Institute, Cary, NC, USA) and R version 4.0.3 (R Foundation for Statistical Computing, Vienna, Austria). For all comparisons, a nominal two-sided alpha error of 0.05 was taken as the threshold for statistical significance.

## Results

### Characteristics of the study population

#### Demographic characteristics

**Fig. 2:**
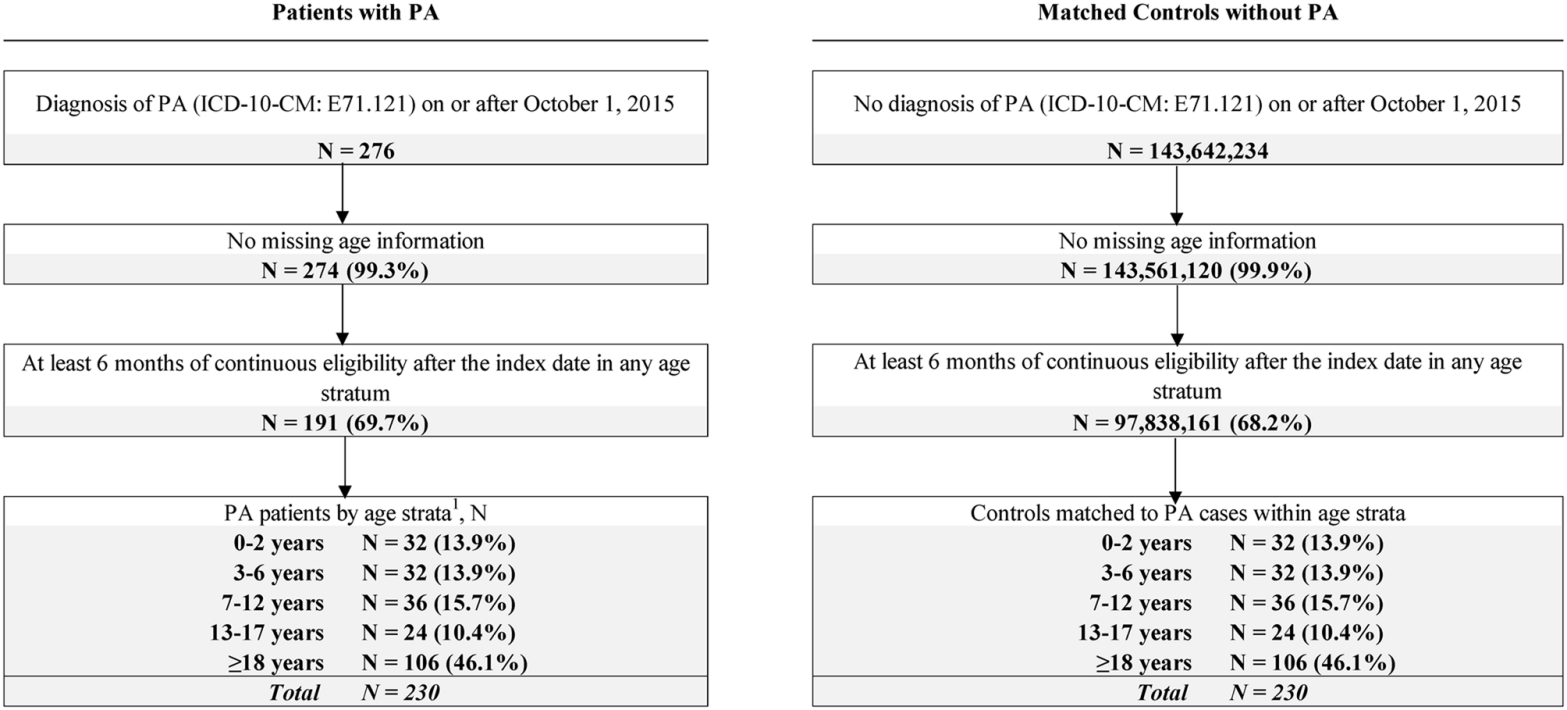
Sample selection diagram. Abbreviations: ICD-10-CM, International Classification of Diseases, Tenth Revision, Clinical Modification; PA, propionic acidemia. Note: [1] As patients with PA could contribute to multiple age strata, the sum of age strata exceeds the total number of patients with PA in the study sample

The study population comprised 191 patients with PA, who contributed 230 observations across ages strata as one patient may contribute to multiple age strata, and 230 non-PA control subjects matched within each age stratum, leading to 230 paired observations across age strata (Fig. 2). Sample sizes ranged from 24 (13–17 years) to 106 matched pairs (≥ 18 years) across age strata. The average age at first observed PA diagnosis was 24.8 years (Table 1). Half of the patients (49.7%) were female and most (91.1%) were commercially or self-insured.

**Table 1:** Demographic and clinical characteristics for patients with PA and matched control subjects without PA

	All patients with PA (earliest age category) *N* = 191	Age stratum									
		0–2 years *N* = 32 pairs		3–6 years *N* = 32 pairs		7–12 years *N* = 36 pairs		13–17 years *N* = 24 pairs		≥ 18 years *N* = 106 pairs	
		PA	Controls	PA	Controls	PA	Controls	PA	Controls	PA	Controls
**Demographic characteristics**											
Age, years	24.8 ± 22.2	0.5 ± 0.6	0.5 ± 0.6	4.0 ± 1.2	4.0 ± 1.1	8.6 ± 1.8	8.6 ± 1.8	14.8 ± 1.7	14.8 ± 1.7	40.6 ± 17.6	40.6 ± 17.6
Female	95 (49.7)	19 (59.4)	19 (59.4)	15 (46.9)	15 (46.9)	10 (27.8)	10 (27.8)	8 (33.3)	8 (33.3)	55 (51.9)	55 (51.9)
Census region											
Midwest	68 (35.6)	14 (43.8)	14 (43.8)	15 (46.9)	15 (46.9)	13 (36.1)	13 (36.1)	10 (41.7)	10 (41.7)	36 (34.0)	37 (34.9)
South	63 (33.0)	14 (43.8)	14 (43.8)	8 (25.0)	8 (25.0)	12 (33.3)	12 (33.3)	4 (16.7)	4 (16.7)	35 (33.0)	35 (33.0)
Northeast	37 (19.4)	3 (9.4)	3 (9.4)	5 (15.6)	5 (15.6)	8 (22.2)	8 (22.2)	4 (16.7)	4 (16.7)	23 (21.7)	23 (21.7)
West	22 (11.5)	1 (3.1)	1 (3.1)	4 (12.5)	4 (12.5)	3 (8.3)	3 (8.3)	6 (25.0)	6 (25.0)	11 (10.4)	11 (10.4)
Unknown	1 (0.5)	0 (0.0)	0 (0.0)	0 (0.0)	0 (0.0)	0 (0.0)	0 (0.0)	0 (0.0)	0 (0.0)	1 (0.9)	0 (0.0)
Index year											
2015	24 (12.6)	4 (12.5)	4 (12.5)	7 (21.9)	7 (21.9)	3 (8.3)	3 (8.3)	5 (20.8)	5 (20.8)	5 (4.7)	5 (4.7)
2016	46 (24.1)	7 (21.9)	7 (21.9)	8 (25.0)	8 (25.0)	13 (36.1)	13 (36.1)	6 (25.0)	6 (25.0)	17 (16.0)	17 (16.0)
2017	15 (7.9)	5 (15.6)	5 (15.6)	1 (3.1)	1 (3.1)	4 (11.1)	4 (11.1)	0 (0.0)	0 (0.0)	9 (8.5)	8 (7.6)
2018	28 (14.7)	4 (12.5)	4 (12.5)	3 (9.4)	4 (12.5)	6 (16.7)	6 (16.7)	1 (4.2)	1 (4.2)	23 (21.7)	24 (22.6)
2019	30 (15.7)	4 (12.5)	4 (12.5)	5 (15.6)	4 (12.5)	3 (8.3)	3 (8.3)	5 (20.8)	5 (20.8)	20 (18.9)	20 (18.9)
2020	27 (14.1)	4 (12.5)	4 (12.5)	5 (15.6)	5 (15.6)	5 (13.9)	5 (13.9)	2 (8.3)	2 (8.3)	19 (17.9)	19 (17.9)
2021	21 (11.0)	4 (12.5)	4 (12.5)	3 (9.4)	3 (9.4)	2 (5.6)	2 (5.6)	5 (20.8)	5 (20.8)	13 (12.3)	13 (12.3)
Flu season (September–March)^1^	122 (63.9)	19 (59.4)	19 (59.4)	20 (62.5)	20 (62.5)	15 (41.7)	15 (41.7)	12 (50.0)	12 (50.0)	61 (57.5)	63 (59.4)
Insurance type											
Commercial/self-insured	174 (91.1)	29 (90.6)	29 (90.6)	32 (100.0)	32 (100.0)	35 (97.2)	35 (97.2)	24 (100.0)	24 (100.0)	93 (87.7)	93 (87.7)
Medicare	11 (5.8)	0 (0.0)	0 (0.0)	0 (0.0)	0 (0.0)	0 (0.0)	0 (0.0)	0 (0.0)	0 (0.0)	11 (10.4)	11 (10.4)
Medicaid	6 (3.1)	3 (9.4)	3 (9.4)	0 (0.0)	0 (0.0)	1 (2.8)	1 (2.78)	0 (0.0)	0 (0.0)	2 (1.9)	2 (1.9)
**Clinical characteristics**											
PA symptoms	108 (56.5)	**22 (68.8)**	**11 (34.4)**	**21 (65.6)**	**5 (15.6)**	12 (33.3)	5 (13.9)	**10 (41.7)**	**1 (4.2)**	**62 (58.5)**	**8 (7.5)**
Anorexia/failure to feed	53 (27.8)	**19 (59.4)**	**8 (25.0)**	**15 (46.9)**	**4 (12.5)**	4 (11.1)	3 (8.3)	**9 (37.5)**	**1 (4.2)**	**17 (16.0)**	**0 (0.0)**
Vomiting	50 (26.2)	**11 (34.4)**	**2 (6.3)**	**16 (50.0)**	**0 (0.0)**	5 (13.9)	2 (5.6)	2 (8.3)	0 (0.0)	**27 (25.5)**	**4 (3.8)**
Metabolic acidosis	50 (26.2)	**13 (40.6)**	**0 (0.0)**	5 (15.6)	0 (0.0)	2 (5.6)	0 (0.0)	3 (12.5)	0 (0.0)	**31 (29.2)**	**0 (0.0)**
Seizures	28 (14.7)	5 (15.6)	1 (3.1)	6 (18.8)	1 (3.1)	**8 (22.2)**	**0 (0.0)**	3 (12.5)	0 (0.0)	**15 (14.2)**	**4 (3.8)**
Hyperammonemia	14 (7.3)	**9 (28.1)**	**0 (0.0)**	4 (12.5)	0 (0.0)	2 (5.6)	0 (0.0)	1 (4.2)	0 (0.0)	2 (1.9)	0 (0.0)
PA-related comorbidities and complications											
Metabolism-related conditions	30 (15.7)	**10 (31.3)**	**0 (0.0)**	**14 (43.8)**	**0 (0.0)**	**6 (16.7)**	**0 (0.0)**	2 (8.3)	0 (0.0)	**6 (5.7)**	**0 (0.0)**
Other BCAA/FA metabolism disorders^2^	13 (6.8)	2 (6.3)	0 (0.0)	**8 (25.0)**	**0 (0.0)**	2 (5.6)	0 (0.0)	1 (4.2)	0 (0.0)	3 (2.8)	0 (0.0)
Methylmalonic acidemia	9 (4.7)	4 (12.5)	0 (0.0)	4 (12.5)	0 (0.0)	2 (5.6)	0 (0.0)	1 (4.2)	0 (0.0)	2 (1.9)	0 (0.0)
Ketonuria	8 (4.2)	2 (6.3)	0 (0.0)	3 (9.4)	0 (0.0)	2 (5.6)	0 (0.0)	0 (0.0)	0 (0.0)	2 (1.9)	0 (0.0)
Hypoglycemia	3 (1.6)	3 (9.4)	0 (0.0)	0 (0.0)	0 (0.0)	0 (0.0)	0 (0.0)	0 (0.0)	0 (0.0)	0 (0.0)	0 (0.0)
Cytopenias	33 (17.3)	**13 (40.6)**	**0 (0.0)**	4 (12.5)	0 (0.0)	1 (2.8)	0 (0.0)	1 (4.2)	0 (0.0)	**21 (19.8)**	**0 (0.0)**
Anemia	26 (13.6)	**10 (31.3)**	**0 (0.0)**	1 (3.1)	0 (0.0)	1 (2.8)	0 (0.0)	1 (4.2)	0 (0.0)	15 (14.2)	0 (0.0)
Neutropenia	6 (3.1)	4 (12.5)	0 (0.0)	2 (6.3)	0 (0.0)	0 (0.0)	0 (0.0)	0 (0.0)	0 (0.0)	0 (0.0)	0 (0.0)
Thrombocytopenia	7 (3.7)	4 (12.5)	0 (0.0)	0 (0.0)	0 (0.0)	0 (0.0)	0 (0.0)	1 (4.2)	0 (0.0)	2 (1.9)	0 (0.0)
Growth complications	31 (16.2)	**14 (43.8)**	**3 (9.4)**	**8 (25.0)**	**0 (0.0)**	**8 (22.2)**	**0 (0.0)**	4 (16.7)	0 (0.0)	**8 (7.5)**	**0 (0.0)**
Growth impairment	26 (13.6)	**10 (31.3)**	**1 (3.1)**	**7 (21.9)**	**0 (0.0)**	**6 (16.7)**	**0 (0.0)**	4 (16.7)	0 (0.0)	**8 (7.5)**	**0 (0.0)**
Failure to thrive	9 (4.7)	6 (18.8)	2 (6.3)	2 (6.3)	0 (0.0)	2 (5.6)	0 (0.0)	0 (0.0)	0 (0.0)	1 (0.9)	0 (0.0)
Cardiac system conditions	17 (8.9)	1 (3.1)	0 (0.0)	1 (3.1)	0 (0.0)	3 (8.3)	0 (0.0)	**6 (25.0)**	**0 (0.0)**	**12 (11.3)**	**0 (0.0)**
Cardiomyopathy	11 (5.8)	0 (0.0)	0 (0.0)	1 (3.1)	0 (0.0)	2 (5.6)	0 (0.0)	4 (16.7)	0 (0.0)	**8 (7.5)**	**0 (0.0)**
Long QT syndrome	6 (3.1)	1 (3.1)	0 (0.0)	0 (0.0)	0 (0.0)	1 (2.8)	0 (0.0)	3 (12.5)	0 (0.0)	4 (3.8)	0 (0.0)
Neurologic and CNS/PNS conditions	64 (33.5)	11 (34.4)	4 (12.5)	**17 (53.1)**	**3 (9.4)**	**16 (44.4)**	**1 (2.8)**	8 (33.3)	2 (8.3)	35 (33.0)	7 (6.6)
Movement disorders	32 (16.8)	**6 (18.8)**	**0 (0.0)**	**8 (25.0)**	**1 (3.1)**	3 (8.3)	0 (0.0)	3 (12.5)	1 (4.2)	**20 (18.9)**	**4 (3.8)**
Delayed development of the speech	16 (8.4)	2 (6.3)	1 (3.1)	9 (28.1)	2 (6.3)	7 (19.4)	1 (2.8)	3 (12.5)	1 (4.2)	**8 (7.5)**	**0 (0.0)**
Intellectual disability	24 (12.6)	4 (12.5)	0 (0.0)	**6 (18.8)**	**0 (0.0)**	5 (13.9)	0 (0.0)	**6 (25.0)**	**0 (0.0)**	**12 (11.3)**	**1 (0.9)**
Psychosis	19 (9.9)	2 (6.3)	0 (0.0)	4 (12.5)	0 (0.0)	5 (13.9)	0 (0.0)	4 (16.7)	0 (0.0)	**11 (10.4)**	**1 (0.9)**
Autism	10 (5.2)	0 (0.0)	0 (0.0)	2 (6.3)	0 (0.0)	4 (11.1)	0 (0.0)	3 (12.5)	0 (0.0)	**6 (5.7)**	**0 (0.0)**
Impaired hearing ability	6 (3.1)	2 (6.3)	2 (6.3)	1 (3.1)	1 (3.1)	0 (0.0)	0 (0.0)	1 (4.2)	0 (0.0)	3 (2.8)	1 (0.9)
Other comorbidities											
Type II diabetes	42 (22.0)	0 (0.0)	0 (0.0)	1 (3.1)	0 (0.0)	1 (2.8)	0 (0.0)	2 (8.3)	0 (0.0)	**38 (35.8)**	**3 (2.8)**
Anxiety	35 (18.3)	0 (0.0)	0 (0.0)	2 (6.3)	0 (0.0)	2 (5.6)	1 (2.8)	5 (20.8)	0 (0.0)	**30 (28.3)**	**16 (15.1)**
Hyperlipidemia	33 (17.3)	0 (0.0)	0 (0.0)	0 (0.0)	0 (0.0)	0 (0.0)	0 (0.0)	1 (4.2)	0 (0.0)	**33 (31.1)**	**20 (18.9)**
Type I diabetes	25 (13.1)	1 (3.1)	0 (0.0)	0 (0.0)	0 (0.0)	5 (13.9)	0 (0.0)	**6 (25.0)**	**0 (0.0)**	**15 (14.2)**	**2 (1.9)**
Obesity	23 (12.0)	2 (6.3)	0 (0.0)	3 (9.4)	0 (0.0)	1 (2.8)	3 (8.3)	0 (0.0)	0 (0.0)	20 (18.9)	11 (10.4)
Depression	21 (11.0)	1 (3.1)	0 (0.0)	1 (3.1)	1 (3.1)	0 (0.0)	1 (2.8)	2 (8.3)	1 (4.2)	**17 (16.0)**	**7 (6.6)**
Asthma	17 (8.9)	2 (6.3)	1 (3.1)	1 (3.1)	2 (6.3)	1 (2.8)	1 (2.8)	3 (12.5)	0 (0.0)	10 (9.4)	3 (2.8)
Epilepsy	16 (8.4)	2 (6.3)	0 (0.0)	5 (15.6)	1 (3.1)	**7 (19.4)**	**0 (0.0)**	1 (4.2)	0 (0.0)	9 (8.5)	2 (1.9)
CKD^3^	14 (7.3)	1 (3.1)	0 (0.0)	1 (3.1)	0 (0.0)	0 (0.0)	0 (0.0)	0 (0.0)	0 (0.0)	**14 (13.2)**	**0 (0.0)**
Thyroid disease	14 (7.3)	1 (3.1)	0 (0.0)	0 (0.0)	0 (0.0)	1 (2.8)	0 (0.0)	3 (12.5)	0 (0.0)	**9 (8.5)**	**2 (1.9)**
Arthritis	11 (5.8)	0 (0.0)	0 (0.0)	0 (0.0)	0 (0.0)	0 (0.0)	0 (0.0)	0 (0.0)	0 (0.0)	11 (10.4)	5 (4.7)
ADHD	6 (3.1)	0 (0.0)	0 (0.0)	2 (6.3)	1 (3.1)	0 (0.0)	2 (5.6)	1 (4.2)	1 (4.2)	4 (3.8)	0 (0.0)
CCI	1.06 ± 1.57	0.19 ± 0.54	0.03 ± 0.18	**0.56 ± 1.08**	**0.06 ± 0.25**	**0.33 ± 0.53**	**0.03 ± 0.17**	**0.92 ± 1.10**	**0.00 ± 0.00**	**1.58 ± 1.92**	**0.17 ± 0.49**
Select conditions^4^											
Diabetes without chronic complication	45 (23.6)	1 (3.1)	0 (0.0)	1 (3.1)	0 (0.0)	4 (11.1)	0 (0.0)	**6 (25.0)**	**0 (0.0)**	**35 (33.0)**	**4 (3.8)**
Chronic pulmonary disease	24 (12.6)	2 (6.3)	1 (3.1)	1 (3.1)	2 (6.3)	1 (2.8)	1 (2.8)	3 (12.5)	0 (0.0)	**17 (16.0)**	**6 (5.7)**
Renal disease	23 (12.0)	1 (3.1)	0 (0.0)	3 (9.4)	0 (0.0)	0 (0.0)	0 (0.0)	2 (8.3)	0 (0.0)	**20 (18.9)**	**0 (0.0)**
Mild liver disease	21 (11.0)	1 (3.1)	0 (0.0)	**8 (25.0)**	**0 (0.0)**	4 (11.1)	0 (0.0)	3 (12.5)	0 (0.0)	**10 (9.4)**	**2 (1.9)**
Congestive heart failure	19 (9.9)	0 (0.0)	0 (0.0)	1 (3.1)	0 (0.0)	2 (5.6)	0 (0.0)	4 (16.7)	0 (0.0)	**16 (15.1)**	**1 (0.9)**
Diabetes with chronic complication	16 (8.4)	0 (0.0)	0 (0.0)	0 (0.0)	0 (0.0)	0 (0.0)	0 (0.0)	1 (4.2)	0 (0.0)	**15 (14.2)**	**2 (1.9)**
Cerebrovascular disease	10 (5.2)	0 (0.0)	0 (0.0)	0 (0.0)	0 (0.0)	0 (0.0)	0 (0.0)	0 (0.0)	0 (0.0)	**11 (10.4)**	**2 (1.9)**
**Treatments**											
Medication/procedure											
L-carnitine	51 (26.7)	**14 (43.8)**	**0 (0.0)**	**14 (43.8)**	**0 (0.0)**	**14 (38.9)**	**0 (0.0)**	**9 (37.5)**	**0 (0.0)**	**19 (17.9)**	**0 (0.0)**
Any antibiotic use	51 (26.7)	12 (37.5)	7 (21.9)	9 (28.1)	7 (21.9)	5 (13.9)	3 (8.3)	**7 (29.2)**	2 (8.3)	26 (24.5)	17 (16.0)
Carglumic acid	4 (2.1)	3 (9.4)	0 (0.0)	1 (3.1)	0 (0.0)	0 (0.0)	0 (0.0)	**1 (4.2)**	0 (0.0)	1 (0.9)	0 (0.0)
Nutritional supplementation	42 (22.0)	**12 (37.5)**	**0 (0.0)**	**8 (25.0)**	**0 (0.0)**	**7 (19.4)**	**0 (0.0)**	**6 (25.0)**	**0 (0.0)**	**20 (18.9)**	**0 (0.0)**
G-tube/NG-tube	32 (16.8)	14 (43.8)	6 (18.8)	**7 (21.9)**	**0 (0.0)**	**7 (19.4)**	**0 (0.0)**	4 (16.7)	0 (0.0)	**10 (9.4)**	**0 (0.0)**
Hemodialysis	10 (5.2)	2 (6.3)	0 (0.0)	2 (6.3)	0 (0.0)	0 (0.0)	0 (0.0)	0 (0.0)	0 (0.0)	**7 (6.6)**	**0 (0.0)**
Peritoneal dialysis, hemofiltration, or other continuous renal replacement therapies	4 (2.1)	2 (6.3)	0 (0.0)	0 (0.0)	0 (0.0)	0 (0.0)	0 (0.0)	0 (0.0)	0 (0.0)	2 (1.9)	0 (0.0)

Data are presented as mean ± standard deviation or n (%). Characteristics were summarized during the 6-month period after the index date. Values in boldface indicate a statistically significant difference between patients with PA and non-PA control subjects (*p* < 0.05)

Abbreviations: ADHD, attention-deficit/hyperactivity disorder; BCAA, branched-chain amino acid; CCI, Charlson Comorbidity Index; CKD, chronic kidney disease; CNS, central nervous system; FA, fatty acid; G-tube, gastronomy tube; NG-tube, nasogastric tube; PA, propionic acidemia; PNS, peripheral nervous system

Notes:

[1] Patients with PA and non-PA control subjects were matched on exposure to flu during flu season (index date between September and March) as this could impact rates of metabolic decompensation

[2] Other disorders of BCAA and FA metabolism included all conditions listed under International Classification of Diseases, 10th Revision, Clinical Modification code E71.x excluding methylmalonic acidemia (E71.120) and PA (E71.121) (see Supplemental Table S1 for codes)

[3] CKD included stage 3–5 disease or ESRD

[4] Conditions with prevalence > 5% in any age stratum are reported

#### Clinical characteristics

Across all age strata, more than half of patients with PA (56.5%) experienced PA symptoms within 6 months of the index date, most commonly anorexia/failure to feed (27.8%), vomiting (26.2%), and metabolic acidosis (26.2%) (Table 1). One-third of patients with PA (33.5%) had a comorbid neurologic or central/peripheral nervous system condition; other frequent comorbidities and complications were cytopenias (17.3%), growth complications (16.2%), and metabolism-related conditions (15.7%).

In general, PA symptoms (e.g., anorexia/failure to feed) and PA-related comorbidities (e.g., metabolism-related conditions, cytopenias, and certain neurologic and central/peripheral nervous system conditions) were more prevalent among younger patients with PA (0–2 and 3–6 years) than in older age strata. On the other hand, older patients with PA were more frequently diagnosed with cardiac system conditions (e.g., cardiomyopathy), psychosis, and autism than younger patients.

Patients with PA more often had PA symptoms and PA-related conditions compared with matched non-PA control subjects in the same age stratum. For example, 68.8% of patients with PA aged 0–2 years and 58.5% of those aged ≥ 18 years had any PA symptoms compared with 34.4% (*p* < 0.05) and 7.6% (*p* < 0.001), respectively, of matched control subjects. Other chronic comorbidities (e.g., type I/II diabetes, anxiety, hyperlipidemia) were also more prevalent among patients with PA.

#### Treatments

Over one-quarter of patients with PA received L-carnitine (26.7%) and nutritional supplementation (22.0%) during the 6-month period after the index date; only a small proportion (2.1%) had recorded carglumic acid use (Table 1). Additionally, 16.8% of patients with PA had used a gastrostomy tube (G-tube)/nasogastric tube and 11.0% (21 patients) had evidence of liver transplantation at any time (18 patients were identified by a diagnosis or procedure code and 3 patients by a treatment proxy). The median age of the earliest observed liver transplantation among the 18 patients with liver transplantation identified by a diagnosis or procedure code– providing a more accurate reflection of transplant age than treatment proxy– was 5.6 years (range: 0.7–49.5 years). Non-PA control subjects had infrequent use of all treatments and procedures examined in this study.

### Rates of MDEs and hospitalizations with PA signs and symptoms in patients with PA

#### MDEs

**Fig. 3:**
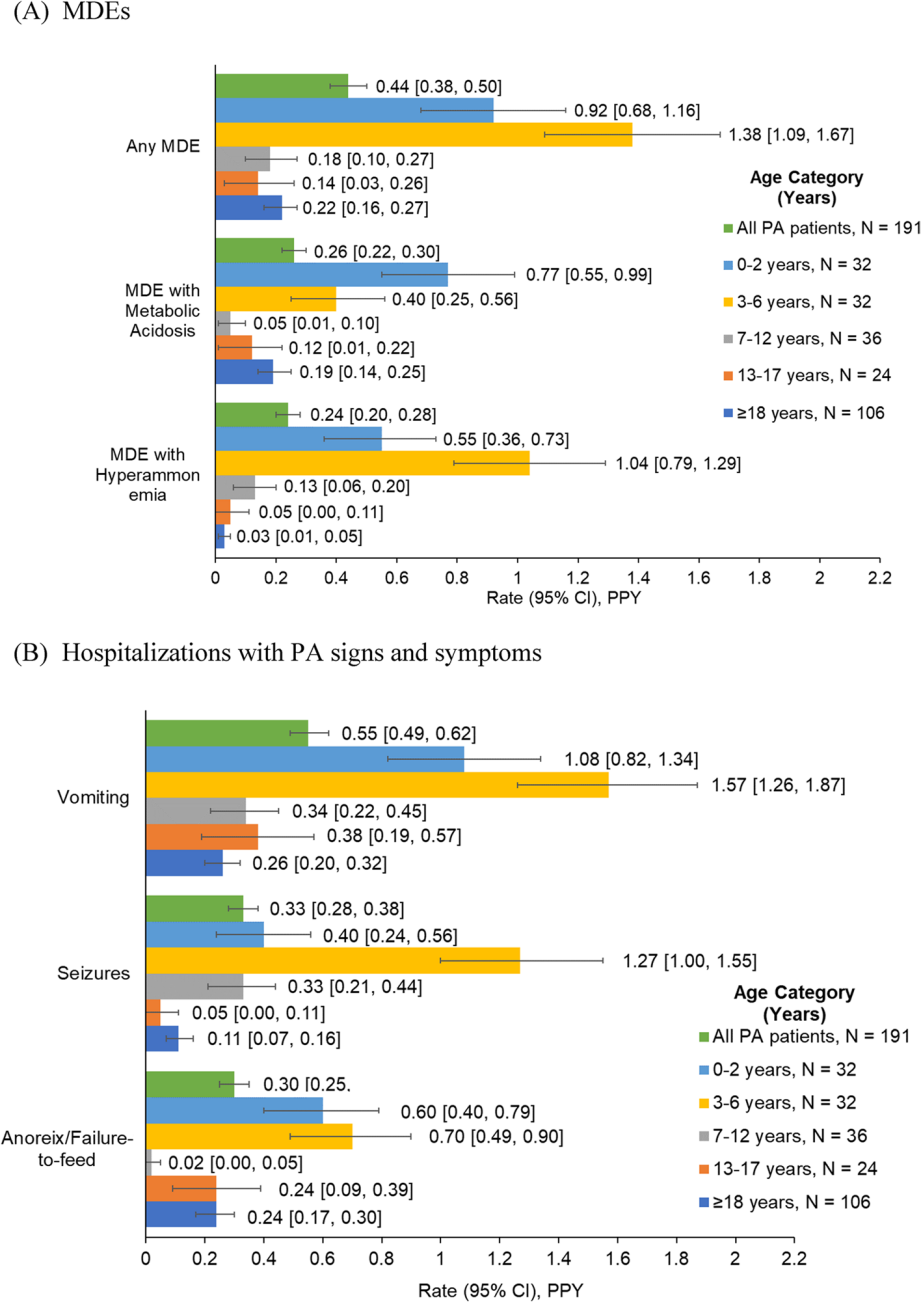
Rates of MDEs [1] and hospitalizations with PA signs and symptoms in patients with PA. Abbreviations: CI, confidence interval; MDE, metabolic decompensation event; PA, propionic acidemia; PPY, per person-year. Note: [1] MDE was defined as a diagnosis of metabolic acidosis and/or hyperammonemia occurring in the inpatient setting. MDE with metabolic acidosis and MDE with hyperammonemia were not mutually exclusive

Over a median (range) follow-up of 2.7 (0.5–6.7) years, 31.4% of patients with PA experienced at least one MDE. The overall rate (95% CI) of MDEs was 0.44 (0.38–0.50) PPY (Fig. 3A), with an average of 8.5 hospital days per MDE (Supplemental Table S1). The rates observed for MDEs presenting with either metabolic acidosis (0.26 [0.22–0.30] PPY) or hyperammonemia (0.24 [0.20–0.28] PPY) were broadly similar, but in the 3–6 year age stratum, MDEs characterized by hyperammonemia appeared to be more common (Fig. 3A).

MDE rates were higher in younger patients than in adults (≥ 18 years). Among patients aged < 18 years, the MDE rate was highest in the 0–2 and 3–6 years age strata (0.92 [0.68–1.16] and 1.38 [1.09–1.67] PPY, respectively) and lowest in the 13–17 years age stratum (0.14 [0.03–0.26] PPY). The rate of MDEs in adults was 0.22 (0.16–0.27) PPY (Fig. 3A).

Consistent results were obtained in a sensitivity analysis of MDEs occurring in either an inpatient or emergency room (> 24 h) setting (i.e., 32.5% of patients experienced any MDEs at a rate of 0.45 [95% CI: 0.39‒0.51] PPY) (Supplemental Figure S1A).

#### Hospitalizations with PA signs and symptoms

Rates of hospitalizations with PA signs and symptoms followed the same trend as MDE rates when considering variation by age strata (Fig. 3B), with vomiting being the most prevalent PA symptom: they were highest in patients with PA aged 0–2 years (with vomiting: 1.08 [0.82–1.34] PPY; with seizures: 0.40 [0.24–0.56] PPY; with anorexia/failure to feed: 0.60 [0.40–0.79] PPY) and 3–6 years (with vomiting: 1.57 [1.26–1.87] PPY; with seizures: 1.27 [1.00–1.55] PPY; with anorexia/failure to feed: 0.70 [0.49–0.90] PPY) and decreased with age. Consistent results were obtained in the sensitivity analysis of PA signs and symptoms occurring in inpatient or emergency room (> 24 h) settings (Supplemental Figure S1B).

### Patient characteristics and clinical outcomes stratified by presence or absence of MDEs

In total, 60 (31.4%) patients with PA experienced at least 1 MDE during the study period; the remaining 131 (68.6%) patients did not experience any MDEs (Table 2). The two cohorts had similar demographic profiles, but patients who had any MDEs more frequently experienced PA symptoms within the 6-month post-index period than those without any MDEs (86.7% vs. 42.8%; *p* < 0.001) and had higher medication use (L-carnitine: 41.7% vs. 19.8%; carglumic acid: 6.7% vs. 0.0%). The difference between cohorts in this symptomatic burden was greatest in the 0–2 years (with vs. without MDE: 87.5% vs. 50.0%; *p* = 0.06), 3–6 years (92.9% vs. 44.4%; *p* < 0.01), and ≥ 18 years (88.2% vs. 44.4%; *p* < 0.001) age strata. PA-related comorbidities and other chronic comorbidities were also more common among patients who had any MDEs than those without, though the differences were not statistically significant in some age strata due to small sample sizes. Liver transplantation, which could occur at any time during a patient’s medical history, was more common among patients who experienced any MDEs (16.7% vs. 8.4%; *p* = 0.15).

**Table 2:** Demographic and clinical characteristics of patients with PA with any MDE vs. no MDE [1]

			Age stratum									
	All patients with PA		0–2 years		3–6 years		7–12 years		13–17 years		≥ 18 years	
	MDE	No MDE	MDE	No MDE	MDE	No MDE	MDE	No MDE	MDE	No MDE	MDE	No MDE
	*N* = 60	*N* = 131	*N* = 16	*N* = 16	*N* = 14	*N* = 18	*N* = 9	*N* = 27	*N* = 6	*N* = 18	*N* = 34	*N* = 72
**Demographic characteristics**												
Age, years	25.2 ± 25.3	24.7 ± 20.7	0.6 ± 0.7	0.4 ± 0.5	3.7 ± 1.2	4.2 ± 1.1	**7.2 ± 0.5**	**9.0 ± 1.9**	**16.2 ± 1.4**	**14.3 ± 1.6**	42.7 ± 20.4	39.7 ± 16.2
Female	26 (43.3)	69 (52.7)	9 (56.3)	10 (62.5)	5 (35.7)	10 (55.6)	2 (22.2)	8 (29.6)	2 (33.3)	6 (33.3)	13 (38.2)	42 (58.3)
Census region												
Midwest	23 (38.3)	45 (34.4)	8 (50.0)	6 (37.5)	7 (50.0)	8 (44.4)	4 (44.4)	9 (33.3)	4 (66.7)	6 (33.3)	11 (32.4)	25 (34.7)
South	19 (31.7)	44 (33.6)	5 (31.3)	9 (56.3)	4 (28.6)	4 (22.2)	3 (33.3)	9 (33.3)	0 (0.0)	4 (22.2)	11 (32.4)	24 (33.3)
Northeast	13 (21.7)	24 (18.3)	3 (18.8)	0 (0.0)	3 (21.4)	2 (11.1)	2 (22.2)	6 (22.2)	2 (33.3)	2 (11.1)	7 (20.6)	16 (22.2)
West	5 (8.3)	17 (13.0)	0 (0.0)	1 (6.3)	0 (0.0)	4 (22.2)	0 (0.0)	3 (11.1)	0 (0.0)	6 (33.3)	5 (14.7)	6 (8.3)
Unknown	0 (0.0)	1 (0.8)	0 (0.0)	0 (0.0)	0 (0.0)	0 (0.0)	0 (0.0)	0 (0.0)	0 (0.0)	0 (0.0)	0 (0.0)	1 (1.4)
Index year												
2015	11 (18.3)	13 (9.9)	4 (25.0)	0 (0.0)	1 (7.1)	6 (33.3)	1 (11.1)	2 (7.4)	**4 (66.7)**	**1 (5.6)**	1 (2.9)	4 (5.6)
2016	12 (20.0)	34 (26.0)	2 (12.5)	5 (31.3)	5 (35.7)	3 (16.7)	1 (11.1)	12 (44.4)	**2 (33.3)**	**4 (22.2)**	5 (14.7)	12 (16.7)
2017	5 (8.3)	10 (7.6)	1 (6.3)	4 (25.0)	0 (0.0)	1 (5.6)	2 (22.2)	2 (7.4)	**0 (0.0)**	**0 (0.0)**	4 (11.8)	5 (6.9)
2018	12 (20.0)	16 (12.2)	3 (18.8)	1 (6.3)	3 (21.4)	0 (0.0)	1 (11.1)	5 (18.5)	**0 (0.0)**	**1 (5.6)**	9 (26.5)	14 (19.4)
2019	7 (11.7)	23 (17.6)	1 (6.3)	3 (18.8)	2 (14.3)	3 (16.7)	1 (11.1)	2 (7.4)	**0 (0.0)**	**5 (27.8)**	7 (20.6)	13 (18.1)
2020	8 (13.3)	19 (14.5)	3 (18.8)	1 (6.3)	2 (14.3)	3 (16.7)	3 (33.3)	2 (7.4)	**0 (0.0)**	**2 (11.1)**	5 (14.7)	14 (19.4)
2021	5 (8.3)	16 (12.2)	2 (12.5)	2 (12.5)	1 (7.1)	2 (11.1)	0 (0.0)	2 (7.4)	**0 (0.0)**	**5 (27.8)**	3 (8.8)	10 (13.9)
Flu season (September–March)^2^	41 (68.3)	81 (61.8)	**13 (81.3)**	**6 (37.5)**	7 (50.0)	13 (72.2)	1 (11.1)	14 (51.9)	**6 (100.0)**	**6 (33.3)**	18 (52.9)	43 (59.7)
Insurance type												
Commercial/self-insured	51 (85.0)	123 (93.9)	15 (93.8)	14 (87.5)	14 (100.0)	18 (100.0)	9 (100.0)	26 (96.3)	6 (100.0)	18 (100.0)	**26 (76.5)**	**67 (93.1)**
Medicare	7 (11.7)	4 (3.1)	0 (0.0)	0 (0.0)	0 (0.0)	0 (0.0)	0 (0.0)	0 (0.0)	0 (0.0)	0 (0.0)	**7 (20.6)**	**4 (5.6)**
Medicaid	2 (3.3)	4 (3.1)	1 (6.3)	2 (12.5)	0 (0.0)	0 (0.0)	0 (0.0)	1 (3.7)	0 (0.0)	0 (0.0)	**1 (2.9)**	**1 (1.4)**
**Clinical characteristics**												
PA symptoms	**52 (86.7)**	**56 (42.7)**	14 (87.5)	8 (50.0)	**13 (92.9)**	**8 (44.4)**	4 (44.4)	8 (29.6)	4 (66.7)	6 (33.3)	**30 (88.2)**	**32 (44.4)**
Metabolic acidosis	**40 (66.7)**	**10 (7.6)**	**12 (75.0)**	**1 (6.3)**	4 (28.6)	1 (5.6)	1 (11.1)	1 (3.7)	**3 (50.0)**	**0 (0.0)**	**24 (70.6)**	**7 (9.7)**
Anorexia/failure to feed	**27 (45.0)**	**26 (19.8)**	**13 (81.3)**	**6 (37.5)**	**10 (71.4)**	**5 (27.8)**	**3 (33.3)**	**1 (3.7)**	4 (66.7)	5 (27.8)	5 (14.7)	12 (16.7)
Vomiting	**26 (43.3)**	**24 (18.3)**	8 (50.0)	3 (18.8)	10 (71.4)	6 (33.3)	3 (33.3)	2 (7.4)	2 (33.3)	0 (0.0)	12 (35.3)	15 (20.8)
Seizures	**14 (23.3)**	**14 (10.7)**	4 (25.0)	1 (6.3)	3 (21.4)	3 (16.7)	4 (44.4)	4 (14.8)	2 (33.3)	1 (5.6)	**9 (26.5)**	**6 (8.3)**
Hyperammonemia	**13 (21.7)**	**1 (0.8)**	**9 (56.3)**	**0 (0.0)**	**4 (28.6)**	**0 (0.0)**	2 (22.2)	0 (0.0)	1 (16.7)	0 (0.0)	1 (2.9)	1 (1.4)
PA-related comorbidities and complications												
Metabolism-related conditions	12 (20.0)	18 (13.7)	6 (37.5)	4 (25.0)	8 (57.1)	6 (33.3)	1 (11.1)	5 (18.5)	1 (16.7)	1 (5.6)	2 (5.9)	4 (5.6)
Ketonuria	5 (8.3)	3 (2.3)	2 (12.5)	0 (0.0)	3 (21.4)	0 (0.0)	0 (0.0)	2 (7.4)	0 (0.0)	0 (0.0)	1 (2.9)	1 (1.4)
Methylmalonic acidemia	4 (6.7)	5 (3.8)	3 (18.8)	1 (6.3)	1 (7.1)	3 (16.7)	0 (0.0)	2 (7.4)	1 (16.7)	0 (0.0)	1 (2.9)	1 (1.4)
Other BCAA/FA metabolism disorders^3^	3 (5.0)	10 (7.6)	1 (6.3)	1 (6.3)	4 (28.6)	4 (22.2)	1 (11.1)	1 (3.7)	0 (0.0)	1 (5.6)	0 (0.0)	3 (4.2)
Hypoglycemia	1 (1.7)	2 (1.5)	1 (6.3)	2 (12.5)	0 (0.0)	0 (0.0)	0 (0.0)	0 (0.0)	0 (0.0)	0 (0.0)	0 (0.0)	0 (0.0)
Cytopenias	**23 (38.3)**	**10 (7.6)**	**11 (68.8)**	**2 (12.5)**	2 (14.3)	1 (5.6)	1 (11.1)	0 (0.0)	1 (16.7)	0 (0.0)	**10 (29.4)**	**7 (9.7)**
Anemia	**19 (31.7)**	**7 (5.3)**	8 (50.0)	2 (12.5)	1 (7.1)	0 (0.0)	1 (11.1)	0 (0.0)	1 (16.7)	0 (0.0)	**10 (29.4)**	**5 (6.9)**
Neutropenia	**5 (8.3)**	**1 (0.8)**	4 (25.0)	0 (0.0)	1 (7.1)	1 (5.6)	0 (0.0)	0 (0.0)	0 (0.0)	0 (0.0)	0 (0.0)	0 (0.0)
Thrombocytopenia	**5 (8.3)**	**2 (1.5)**	4 (25.0)	0 (0.0)	0 (0.0)	0 (0.0)	0 (0.0)	0 (0.0)	1 (16.7)	0 (0.0)	0 (0.0)	2 (2.8)
Growth complications	**15 (25.0)**	**16 (12.2)**	8 (50.0)	6 (37.5)	5 (35.7)	3 (16.7)	3 (33.3)	5 (18.5)	**3 (50.0)**	**1 (5.6)**	4 (11.8)	4 (5.6)
Growth impairment	12 (20.0)	14 (10.7)	5 (31.3)	5 (31.3)	5 (35.7)	2 (11.1)	3 (33.3)	3 (11.1)	**3 (50.0)**	**1 (5.6)**	4 (11.8)	4 (5.6)
Failure to thrive	**7 (11.7)**	**2 (1.5)**	5 (31.3)	1 (6.3)	1 (7.1)	1 (5.6)	0 (0.0)	2 (7.4)	0 (0.0)	0 (0.0)	1 (2.9)	0 (0.0)
Cardiac system conditions	**12 (20.0)**	**5 (3.8)**	1 (6.3)	0 (0.0)	0 (0.0)	1 (5.6)	1 (11.1)	2 (7.4)	**5 (83.3)**	**1 (5.6)**	**9 (26.5)**	**3 (4.2)**
Cardiomyopathy	**7 (11.7)**	**4 (3.1)**	0 (0.0)	0 (0.0)	0 (0.0)	1 (5.6)	0 (0.0)	2 (7.4)	**3 (50.0)**	**1 (5.6)**	**6 (17.6)**	**2 (2.8)**
Long QT syndrome	**5 (8.3)**	**1 (0.8)**	1 (6.3)	0 (0.0)	0 (0.0)	0 (0.0)	1 (11.1)	0 (0.0)	2 (33.3)	1 (5.6)	3 (8.8)	1 (1.4)
Neurologic and CNS/PNS conditions	**31 (51.7)**	**33 (25.2)**	6 (37.5)	5 (31.3)	10 (71.4)	7 (38.9)	6 (66.7)	10 (37.0)	4 (66.7)	4 (22.2)	**19 (55.9)**	**16 (22.2)**
Movement disorders	15 (25.0)	17 (13.0)	4 (25.0)	2 (12.5)	4 (28.6)	4 (22.2)	0 (0.0)	3 (11.1)	1 (16.7)	2 (11.1)	**11 (32.4)**	**9 (12.5)**
Intellectual disability	**13 (21.7)**	**11 (8.4)**	3 (18.8)	1 (6.3)	4 (28.6)	2 (11.1)	2 (22.2)	3 (11.1)	3 (50.0)	3 (16.7)	7 (20.6)	5 (6.9)
Psychosis	**12 (20.0)**	**7 (5.3)**	2 (12.5)	0 (0.0)	3 (21.4)	1 (5.6)	2 (22.2)	3 (11.1)	2 (33.3)	2 (11.1)	**7 (20.6)**	**4 (5.6)**
Delayed development of the speech	6 (10.0)	10 (7.6)	1 (6.3)	1 (6.3)	6 (42.9)	3 (16.7)	2 (22.2)	5 (18.5)	0 (0.0)	3 (16.7)	3 (8.8)	5 (6.9)
Autism	4 (6.7)	6 (4.6)	0 (0.0)	0 (0.0)	1 (7.1)	1 (5.6)	1 (11.1)	3 (11.1)	1 (16.7)	2 (11.1)	3 (8.8)	3 (4.2)
Impaired hearing ability	3 (5.0)	3 (2.3)	0 (0.0)	2 (12.5)	1 (7.1)	0 (0.0)	0 (0.0)	0 (0.0)	1 (16.7)	0 (0.0)	2 (5.9)	1 (1.4)
Other comorbidities												
Type II diabetes	15 (25.0)	27 (20.6)	0 (0.0)	0 (0.0)	0 (0.0)	1 (5.6)	0 (0.0)	1 (3.7)	0 (0.0)	2 (11.1)	15 (44.1)	23 (31.9)
Hyperlipidemia	14 (23.3)	19 (14.5)	0 (0.0)	0 (0.0)	0 (0.0)	0 (0.0)	0 (0.0)	0 (0.0)	1 (16.7)	0 (0.0)	14 (41.2)	19 (26.4)
Anxiety	13 (21.7)	22 (16.8)	0 (0.0)	0 (0.0)	0 (0.0)	2 (11.1)	0 (0.0)	2 (7.4)	1 (16.7)	4 (22.2)	13 (38.2)	17 (23.6)
Depression	**12 (20.0)**	**9 (6.9)**	1 (6.3)	0 (0.0)	0 (0.0)	1 (5.6)	0 (0.0)	0 (0.0)	2 (33.3)	0 (0.0)	9 (26.5)	8 (11.1)
Obesity	11 (18.3)	12 (9.2)	2 (12.5)	0 (0.0)	2 (14.3)	1 (5.6)	1 (11.1)	0 (0.0)	0 (0.0)	0 (0.0)	8 (23.5)	12 (16.7)
Asthma	8 (13.3)	9 (6.9)	1 (6.3)	1 (6.3)	1 (7.1)	0 (0.0)	0 (0.0)	1 (3.7)	2 (33.3)	1 (5.6)	4 (11.8)	6 (8.3)
CKD^4^	**8 (13.3)**	**6 (4.6)**	0 (0.0)	1 (6.3)	1 (7.1)	0 (0.0)	0 (0.0)	0 (0.0)	0 (0.0)	0 (0.0)	**9 (26.5)**	**5 (6.9)**
Type I diabetes	6 (10.0)	19 (14.5)	0 (0.0)	1 (6.3)	0 (0.0)	0 (0.0)	0 (0.0)	5 (18.5)	0 (0.0)	6 (33.3)	6 (17.6)	9 (12.5)
Epilepsy	6 (10.0)	10 (7.6)	2 (12.5)	0 (0.0)	2 (14.3)	3 (16.7)	3 (33.3)	4 (14.8)	0 (0.0)	1 (5.6)	**6 (17.6)**	**3 (4.2)**
Thyroid disease	6 (10.0)	8 (6.1)	0 (0.0)	1 (6.3)	0 (0.0)	0 (0.0)	0 (0.0)	1 (3.7)	2 (33.3)	1 (5.6)	4 (11.8)	5 (6.9)
Arthritis	4 (6.7)	7 (5.3)	0 (0.0)	0 (0.0)	0 (0.0)	0 (0.0)	0 (0.0)	0 (0.0)	0 (0.0)	0 (0.0)	4 (11.8)	7 (9.7)
ADHD	3 (5.0)	3 (2.3)	0 (0.0)	0 (0.0)	2 (14.3)	0 (0.0)	0 (0.0)	0 (0.0)	0 (0.0)	1 (5.6)	1 (2.9)	3 (4.2)
CCI	**1.7 ± 2.1**	**0.8 ± 1.2**	0.1 ± 0.3	0.3 ± 0.7	0.9 ± 1.5	0.3 ± 0.6	0.2 ± 0.4	0.4 ± 0.6	**2.0 ± 1.6**	**0.6 ± 0.6**	**2.7 ± 2.3**	**1.1 ± 1.5**
Select conditions^5^												
Diabetes without chronic complication	15 (25.0)	30 (22.9)	0 (0.0)	1 (6.3)	0 (0.0)	1 (5.6)	0 (0.0)	4 (14.8)	0 (0.0)	6 (33.3)	15 (44.1)	20 (27.8)
Renal disease	**14 (23.3)**	**9 (6.9)**	0 (0.0)	1 (6.3)	3 (21.4)	0 (0.0)	0 (0.0)	0 (0.0)	2 (33.3)	0 (0.0)	12 (35.3)	8 (11.1)
Chronic pulmonary disease	12 (20.0)	12 (9.2)	1 (6.3)	1 (6.3)	1 (7.1)	0 (0.0)	0 (0.0)	1 (3.7)	2 (33.3)	1 (5.6)	8 (23.5)	9 (12.5)
Congestive heart failure	**12 (20.0)**	**7 (5.3)**	0 (0.0)	0 (0.0)	0 (0.0)	1 (5.6)	0 (0.0)	2 (7.4)	**3 (50.0)**	**1 (5.6)**	**11 (32.4)**	**5 (6.9)**
Diabetes with chronic complication	**10 (16.7)**	**6 (4.6)**	0 (0.0)	0 (0.0)	0 (0.0)	0 (0.0)	0 (0.0)	0 (0.0)	0 (0.0)	1 (5.6)	**10 (29.4)**	**5 (6.9)**
Mild liver disease	9 (15.0)	12 (9.2)	1 (6.3)	0 (0.0)	4 (28.6)	4 (22.2)	1 (11.1)	3 (11.1)	2 (33.3)	1 (5.6)	5 (14.7)	5 (6.9)
Cerebrovascular disease	3 (5.0)	7 (5.3)	0 (0.0)	0 (0.0)	0 (0.0)	0 (0.0)	0 (0.0)	0 (0.0)	0 (0.0)	0 (0.0)	4 (11.8)	7 (9.7)
**Treatments**												
Medication/procedure												
L-carnitine	**25 (41.7)**	**26 (19.8)**	**11 (68.8)**	**3 (18.8)**	9 (64.3)	5 (27.8)	**7 (77.8)**	**7 (25.9)**	**6 (100.0)**	**3 (16.7)**	6 (17.6)	13 (18.1)
Any antibiotic use	**25 (41.7)**	**26 (19.8)**	8 (50.0)	4 (25.0)	5 (35.7)	4 (22.2)	2 (22.2)	3 (11.1)	**5 (83.3)**	**2 (11.1)**	10 (29.4)	16 (22.2)
Carglumic acid	**4 (6.7)**	**0 (0.0)**	3 (18.8)	0 (0.0)	1 (7.1)	0 (0.0)	0 (0.0)	0 (0.0)	1 (16.7)	0 (0.0)	1 (2.9)	0 (0.0)
Nutritional supplementation	**21 (35.0)**	**21 (16.0)**	9 (56.3)	3 (18.8)	6 (42.9)	2 (11.1)	3 (33.3)	4 (14.8)	**4 (66.7)**	**2 (11.1)**	8 (23.5)	12 (16.7)
G-tube/NG-tube	**20 (33.3)**	**12 (9.2)**	**10 (62.5)**	**3 (18.8)**	5 (35.7)	2 (11.1)	**5 (55.6)**	**2 (7.4)**	2 (33.3)	2 (11.1)	5 (14.7)	5 (6.9)
Hemodialysis	**7 (11.7)**	**3 (2.3)**	1 (6.3)	1 (6.3)	2 (14.3)	0 (0.0)	0 (0.0)	0 (0.0)	0 (0.0)	0 (0.0)	**5 (14.7)**	**2 (2.8)**
Peritoneal dialysis, hemofiltration, or other continuous renal replacement therapies	3 (5.0)	1 (0.8)	1 (6.3)	1 (6.3)	0 (0.0)	0 (0.0)	0 (0.0)	0 (0.0)	0 (0.0)	0 (0.0)	2 (5.9)	0 (0.0)

Data are presented as mean ± standard deviation or n (%). Characteristics were summarized during the 6-month period after the index date. Values in boldface indicate a statistically significant difference between patients with PA and non-PA control subjects (*p* < 0.05)

Abbreviations: ADHD, attention-deficit/hyperactivity disorder; BCAA, branched-chain amino acid; CCI, Charlson Comorbidity Index; CKD, chronic kidney disease; CNS, central nervous system; FA, fatty acid; G-tube, gastronomy tube; MDE, metabolic decompensation event; NG-tube, nasogastric tube; PA, propionic acidemia; PNS, peripheral nervous system

Notes:

[1] MDEs were defined as a diagnosis of metabolic acidosis or hyperammonemia made in the inpatient setting

[2] Patients with PA with MDEs and those without MDEs were matched on exposure to flu during flu season (index date between September and March) as this could impact rates of metabolic decompensation

[3] Other disorders of BCAA and FA metabolism included all conditions listed under International Classification of Diseases, 10th Revision, Clinical Modification code E71.x excluding methylmalonic acidemia (E71.120) and PA (E71.121)

[4] CKD included stage 3–5 disease or ESRD

[5] Conditions with prevalence > 5% in any age stratum are reported

Irrespective of whether they experienced MDEs or not, patients with PA had a higher frequency of PA-related symptoms, PA-related conditions, and other chronic comorbidities than matched non-PA control subjects (Supplemental Tables S2 and S3). In the 0–2 years age stratum, PA symptoms were observed in 87.5% of patients with any MDEs vs. 43.8% of matched non-PA control subjects (*p* < 0.05), and in 50.0% of patients without any MDEs vs. 25.0% of matched non-PA control subjects (*p* = 0.386). Significant differences in PA symptoms were observed between adult patients with PA, with or without MDEs, and their matched control subjects.

**Fig. 4:**
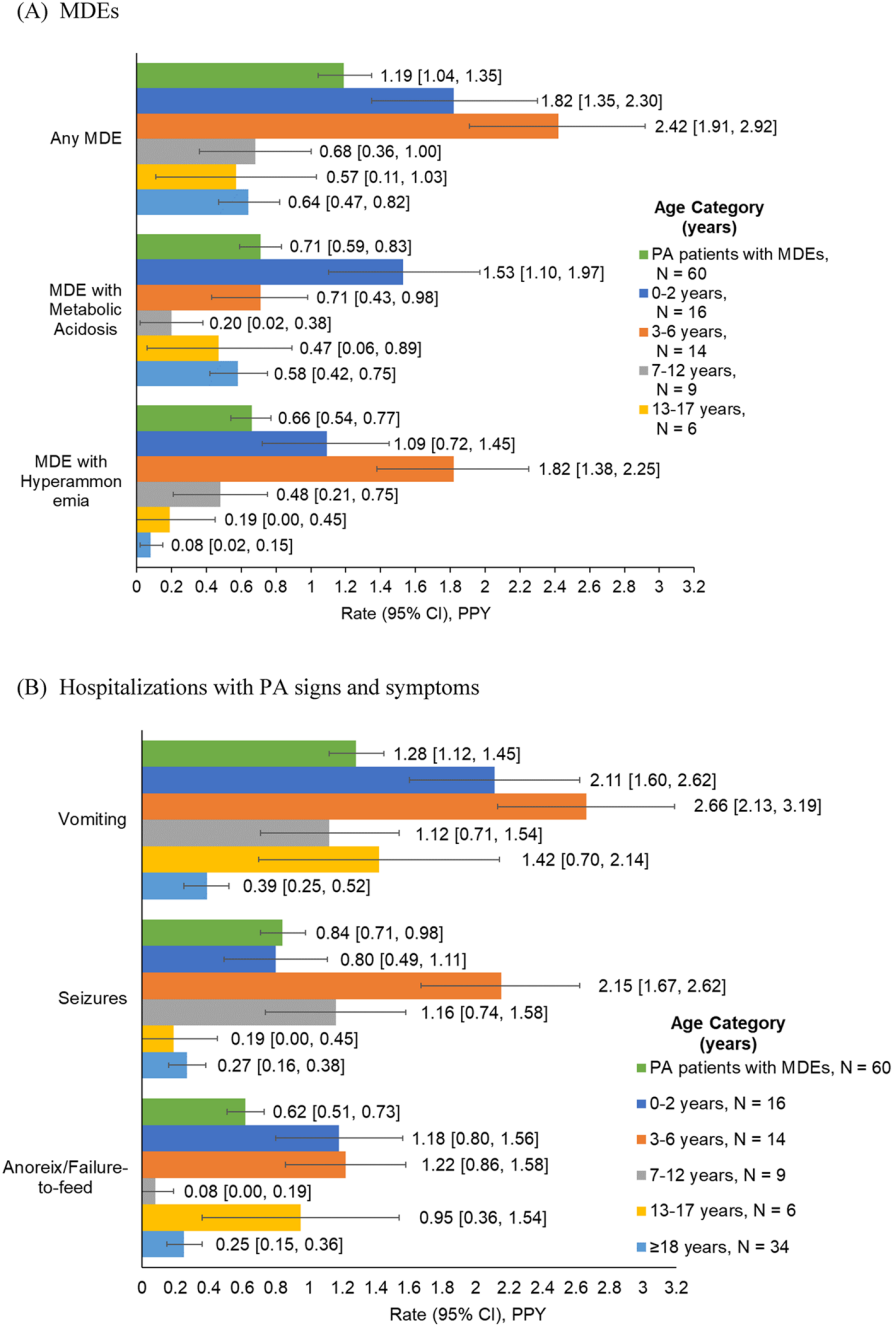
Rates of MDEs [1] and hospitalizations with PA signs and symptoms in patients with PA with any MDE. Abbreviations: CI, confidence interval; MDE, metabolic decompensation event; PA, propionic acidemia; PPY, per person-year. Note: [1] MDE was defined as a diagnosis of metabolic acidosis or hyperammonemia occurring in the inpatient setting

Overall, in patients with PA who experienced any MDEs, the rate (95% CI) of MDEs was 1.19 (1.04–1.35) PPY over a median follow-up of 2.7 years (Fig. 4A). Among patients aged < 18 years, the MDE rate was highest at 3–6 years (2.42 [1.91–2.92] PPY) followed by 0–2 years (1.82 [1.04–1.35] PPY), and lowest at 13–17 years (0.57 [0.11–1.03] PPY); in adults, the rate was lower than in children, but slightly higher than in adolescents (0.64 [0.47–0.82] PPY). Rates of hospitalizations with PA signs and symptoms were also highest at 3–6 years (vomiting: 2.66 [2.13–3.19] PPY; seizures: 2.15 [1.67–2.62] PPY; anorexia/failure to feed: 1.22 [0.86–1.58] PPY) and were generally lower in adults than in younger patients (Fig. 4B).

### MDE rates before vs. after the start of the COVID-19 pandemic

Among the 191 patients with PA, 140 (73.3%) had at least 6 months of follow-up before the start of the COVID-19 pandemic and 119 (62.3%) had at least 6 months of follow-up after. The MDE rate was lower after the start of the pandemic (0.32 [0.24–0.40] PPY) than pre-pandemic (0.51 [0.43–0.59] PPY), indicating a 37% reduction (Supplemental Figure S2A). In both time periods, MDE rates were higher in patients aged < 18 years than in adults. Similarly, rates of hospitalizations with PA signs and symptoms were lower after the pandemic started (vomiting: 0.39 [0.30–0.47] PPY; seizures: 0.26 [0.19–0.33] PPY; anorexia/failure to feed: 0.16 [0.10–0.21] PPY) compared with the pre–COVID-19 period (vomiting: 0.66 [0.57–0.75] PPY; seizures: 0.37 [0.30–0.44] PPY; anorexia/failure to feed: 0.40 [0.32–0.47] PPY), with higher rates in younger patients than in adults in both periods (Supplemental Figure S2B).

## Discussion

Patients with PA experience recurrent life-threatening MDEs and often have multisystemic complications, however, there is limited evidence quantifying the incremental burden of PA compared to the general population in a large and representative population. With this in view, this study is the largest and first using a US health insurance claims database to describe the characteristics and clinical outcomes of patients with PA and matched non-PA control subjects– stratified by age. The results revealed that patients with PA had substantially higher rates of MDEs, hospitalizations with PA signs and symptoms, and comorbidities across all age strata compared to matched non-PA control subjects. MDEs and some PA-related complications such as metabolism-related conditions, growth complications, and cytopenias were more frequent in younger vs. older patients with PA, whereas the latter had higher rates of cardiovascular and certain neurologic manifestations. Patients with PA who experienced at least 1 MDE (nearly one-third of our PA cohort), which is a particularly challenging population that encountered hospitalizations and severe symptoms, had significantly higher rates of PA symptoms and comorbidities than those without any MDEs. Further, patients with MDEs had higher use of treatments, such as L-carnitine and carglumic acid compared to patients without MDEs. Nevertheless, both patient subgroups had more comorbidities than matched subjects without PA. This indicates that the clinical burden of PA is driven by multiple types of comorbidities and complications, not only MDEs. Taken together, these findings provide a benchmark for future studies on patients with PA and highlight both the disease burden and unmet therapeutic needs of this population.

The phenotypic spectrum of PA is broad and the features differentiating degrees of disease severity (i.e., symptoms and biochemical indices such as residual PCC activity) are not well defined [36]; in some cases, patients may be asymptomatic despite a diagnosis of PA based on family history or newborn screening [37, 38]. To obtain a more nuanced picture of the clinical presentation on a large sample, the current study compared characteristics of patients with PA and non-PA control subjects within different age strata. Only a few previous studies have described the clinical features of patients with PA at specific ages and most did not perform analyses by age because of small sample sizes [30, 37, 39, 40]. An exception is a 2012 survey of 58 patients with PA in the US (age: 3 months to 33 years) conducted by the Propionic Acidemia Foundation (PAF), in which several complications (e.g., cardiac arrhythmia) were reported to be more prevalent in the 6–17 years age category than in the other age categories (≤ 5 years and ≥ 18 years) [39]. The present study is one of the first studies and the largest to date to analyze clinical characteristics and outcomes of patients with PA stratified by age. Consistent with the observation that neonates exhibit the most severe manifestation of the disease [41], there was a higher prevalence of PA symptoms, PA-related metabolic comorbidities, and other complications including some neurologic conditions among younger patients with PA (up to 6 years of age) than in older age strata. Meanwhile, cardiomyopathy, psychosis, and autism were more common in older patients with PA. These results reinforce other literature that the clinical presentation of PA and therefore the natural history per se, can differ according to age, which has important implications for disease management and highlights the challenges in predicting the disease course, emphasizing the urgent need for treatments aimed at preventing disease worsening.

Interestingly, the prevalence of certain PA-related complications was lower in our cohort than in other studies. For example, this study found lower rates of seizure (15% vs. 41%) and anemia (14% vs. 32%) compared to the PAF survey study [39]. A European chart review study of 55 patients (age range, 5 days–19 years) also reported higher frequencies of anemia (> 80%), psychomotor retardation (55% vs. 17% with movement disorders in our cohort), and delayed speech development (55% vs. 8%).^38^ The lower rates observed in our study may reflect the shorter 6-months assessment period (vs. entire medical history in the other studies) and limited availability of data typically available in unstructured fields, such as seizure records. It is possible these prior studies included more severe patients (e.g., from centers of excellence) compared to the patient population from a large representative insurance claims database. Because we selected patients using a diagnosis code for PA irrespective of disease severity, disease manifestation, and time of onset, the study population would be more representative of the entirety of the US PA population, which includes patients with mild or no symptoms (9-17% of the PA population) [42] who did not receive treatment or experience clinical complications. Additionally, this study included mostly commercially insured patients, which may present with a milder form of disease. These factors may collectively lead to a lower rate of PA complications as compared to studies that enrolled more severe patients. Supporting this possibility, just 27% of patients received L-carnitine and 2.1% received carglumic acid treatment, compared with > 90% of patients in other studies [30, 37, 38]. 

In the Mapping the Patient Journey in MMA and PA (MaP) observational study of 50 patients in North America and Europe with methylmalonic acidemia or PA (NCT03484767), nearly all of whom had MDEs (98%), 78% had their first MDE by the age of 3 months [43]. These patients had a higher annualized MDE frequency than those who experienced first MDE after 3 months of age. Such studies are important, but there may be a selection bias as almost all patients whose records were abstracted, had MDEs, while the literature has shown that not all patients with PA experience MDEs.

In the current study, nearly one-third of all patients with PA experienced at least 1 MDE in an inpatient setting, with hyperammonemia (55%) and metabolic acidosis (59%) present in most events. In a series of PA cases treated at a single center in the US (age range, 0–28 years), 38% had a history of any MDEs [37]; in a chart review study of 20 Amish patients with PA, 35% were hospitalized for MDEs [40]. The consistency in the proportion of patients with MDEs across studies supports the robustness of the claims-based algorithm that we used to identify MDEs in the claims data– another unique contribution of this paper– supporting use of claims as a potential source for future research in PA.

In contrast to prior literature that suggest that the MDE rates are highest in the first year after birth and decline thereafter [31, 38, 44], this study observed higher rates of MDE and hospitalization with PA signs and symptoms (vomiting, seizures, and failure to feed) in patients aged 3–6 years than in those aged 0–2 years, which might be due to the more stringent monitoring for MDE prevention and better controlled diet in the younger age stratum. Nevertheless, consistent with prior literature, MDE rates in this study declined with age after year 6, with the lowest rates in the 13–17 years age stratum. However, we observed that MDE rates increased again in patients ≥ 18 years. This is the first report of a U-shaped relationship between MDE occurrence and patient age. Although the pathophysiologic basis for this trend is unclear, it provides further evidence for age-dependent differences (or changes) in the clinical presentation of PA, with MDE rates generally higher in younger children than in older groups.

We also observed that the prevalence of PA symptoms, neurologic conditions, and cardiac system conditions in the 6-month post-index period were 2 to 5 times higher in patients with PA with MDEs vs. without any MDEs. This is in line with the finding from the aforementioned case series that patients with PA who experienced MDEs had more severe complications (including cardiovascular and neurologic abnormalities) and often required medical interventions such as G-tube feedings and hemofiltration than those without MDEs [37]. Thus, the presence of MDEs may reflect a more severe phenotype that predicts worse outcomes. In fact, the number of MDEs was shown to be negatively correlated with intelligence quotient (IQ) in 40 patients with PA between 2.5 and 18 years of age although notably, there was no correlation between patients’ IQ and their age or residual PCC activity [44]. In the Egyptian cohort, patients aged > 2 years who did not require intensive care had a higher IQ than those who were admitted to the ICU [31]. Although these findings need to be validated in larger samples, they suggest that preventing or reducing the frequency of MDEs in the early postnatal period can lead to better neurocognitive outcomes in later life. Moreover, although we and others observed that MDE rates decline with age in patients with PA, given the severity of neurologic and other complications that can develop over time, treating patients immediately following diagnosis at infancy should be a primary goal in the long-term management plan for PA.

As the study period encompassed the COVID-19 pandemic, we conducted stratified analyses to assess its impact on rates of MDEs and hospitalization with PA signs and symptoms. MDE rates were consistently higher in patients < 18 years of age than in adults in both the pre– and post–COVID-19 periods. Rates of MDEs and hospitalization with PA signs and symptoms declined after the start of the pandemic (March 2020), possibly due to reduced exposure from social isolation measures in the US, which in turn would reduce triggers such as infections, injuries, or physical activities, thereby lowering the risk of catabolism and MDEs during the lockdown period.

Liver transplantation is a non-curative treatment option for patients with severe and persistent PA symptoms [20, 45]. Unlike medical nutrition therapy, supplements, and conventional pharmacotherapies that offset or reverse the effects of PCC enzyme deficiency, transplantation targets the underlying cause of the disease by restoring functional PCC enzyme in the liver [41]. The clinical benefit from liver transplantation in patients with PA is established. A systematic review and meta-analysis of 21 studies with 70 patients showed that liver transplantation led to metabolic stabilization, reversed preexisting cardiomyopathy, and improved neurodevelopmental deficits, with acceptable patient and allograft survival rates [19]. In our study, 21 patients (11.0%) underwent liver transplantation at a median age of 5.6 years. In other US studies, the age of patients at the time of transplantation ranged from < 1 year to > 21 years [46, 47]. Frequent metabolic decompensations are a common indication for liver transplantation in patients with PA, and most studies have reported no occurrences of MDEs after this treatment [21, 22, 46–48]. In the MaP study, the mean annualized MDE rate was approximately 50% lower in patients who underwent liver transplantation compared with patients without a transplant, although liver transplantation does not completely eliminate the occurrence of MDEs [43]. Despite the effectiveness of this treatment modality in PA, organ transplantation is not without drawbacks such as high cost, donor availability, the requirement for ongoing monitoring and maintenance therapy following the procedure leading to decreased quality of life, and the risks inherent to any surgery and organ transplantation such as infection and lifelong immune suppression, and in the worst case– death [41, 45]. Therefore, less invasive therapeutic strategies involving functional restoration of PCC enzyme are needed to alleviate severe complications of the disease and improve overall quality of life of patients and caregivers.

### Strengths and limitations

This study examined the characteristics and outcomes of the largest contemporary cohort of patients with PA in the US analyzed to date. The number of beneficiaries covered by the IQVIA PharMetrics Plus Claims database enabled selection of a large and diverse sample of real-world patients along with non-PA control subjects matched on similar demographic characteristics within each age stratum, allowing for accurate assessment of disease burden at different ages. All healthcare encounters covered by the insurance provider were available from the closed claims.

There were also limitations to this study. First, the sample size was relatively small within a given age stratum or in stratified analyses, limiting our ability to conduct adjusted statistical comparison or to conduct analyses before vs. after therapy initiation. Second, due to the left-truncated nature of claims data and unavailability of the ICD-10-CM diagnosis code for PA before 2015, the initial diagnosis of PA may not have been visible, limiting the ability to assess clinical burden from PA onset. This also limited our ability to understand if patients were diagnosed with PA during a newborn screening program during our study window. Third, mortality data are not available, and comorbidities, treatments, and clinical outcomes may have been misclassified or under-reported if associated codes are not present in claims. Furthermore, an assessment of pre- vs. post-transplantation outcomes was not feasible due to the small sample size and lack of pre- vs. post- transplantation follow-up time. Finally, the results of this study may not be generalizable to the whole PA population in the US as the majority of patients were covered by commercial insurance; in particular, pediatric patients with Medicaid coverage (whose socioeconomic status may differ from that of commercially insured patients) were not fully captured.

## Conclusions

With data over a median 2.7 years of follow-up, this first-of-its-kind large contemporary study using a representative US health insurance claims database showed that patients with PA experience a substantial burden related to their disease compared with matched control subjects without PA, with high rates of MDEs and high prevalence of PA symptoms and comorbidities, in patients with and without MDEs. The study also carefully examined each age stratum and demonstrated that the clinical burden, including MDE rates, is higher in younger patients (≤ 6 years old). In addition, the clinical burden was greater in patients with PA who experienced MDEs compared to those who did not experience any MDEs; however, both groups of patients experienced a notable incremental burden compared to patients without PA. Taken together, the findings point to the substantial clinical burden of PA in patients of all ages, with or without MDEs, and underscore the urgent need for improved clinical outcomes in these patients.

## Electronic supplementary material

Below is the link to the electronic supplementary material.

Supplementary Material 1

## Data Availability

The datasets generated and analyzed in this study are not publicly available as they were used pursuant to a data use agreement. The data are available through requests made directly to IQVIA.

## Abbreviations

ACMG: American College of Medical Genetics and Genomics
ADHD: Attention-deficit/hyperactivity: disorder
BCAA: Branched-chain amino acid
CCI: Charlson Comorbidity Index
CI: Confidence intervals
CKD: Chronic kidney disease
CNS: Central nervous system
CoA: Coenzyme A
HIPAA: Health Insurance Portability and Accountability Act
IEM: Inborn errors of metabolism
IQ: Intelligence quotient
IQR: Interquartile range
MDE: Metabolic decompensation events
NDC: National Drug Code
PA: Propionic acidemia
PAF: Propionic Acidemia Foundation
PCC: Propionyl coenzyme A carboxylase
PNS: Peripheral nervous system
PPY: Per patient-year
SD: Standard deviation
TCA: Tricarboxylic acid
US: United States
