# Supplementary material for: Clinical burden of propionic acidemia in the United States: a claims-based study by age stratum
Source: Orphanet J Rare Dis. 2025 Jul 15;20:362. doi: 10.1186/s13023-025-03901-2 (PMC12265150; doi:10.1186/s13023-025-03901-2)
Supplement: Supplementary file 1 — Supplementary Material 1 [file 13023_2025_3901_MOESM1_ESM.docx]

# SUPPLEMENTAL MATERIAL

## Supplemental Table S1. Characteristics of MDEs^1^ and hospitalizations with PA signs and symptoms in patients with PA

|  | **All patients with PA** | **Age stratum** | | | | |
| --- | --- | --- | --- | --- | --- | --- |
|  |  | **0–2 years** | **3–6 years** | **7–12 years** | **13–17 years** | **≥18 years** |
|  | **N=191** | **N=32** | **N=32** | **N=36** | **N=24** | **N=106** |
| **MDE** |  |  |  |  |  |  |
| Any MDE |  |  |  |  |  |  |
| Number of patients with event during follow-up | 60 (31.4) | 15 (46.9) | 9 (28.1) | 6 (16.7) | 5 (20.8) | 32 (30.2) |
| Length of stay (days), per-patient-per-MDE |  |  |  |  |  |  |
| Mean±SD | 8.54±9.15 | 11.88±7.49 | 4.43±2.11 | 3.18±0.77 | 5.90±2.46 | 9.41±11.46 |
| Median | 5.00 | 13.00 | 4.00 | 3.00 | 7.00 | 4.50 |
| IQR | (3.00, 12.16) | (4.86, 15.24) | (3.00, 5.11) | (3.00, 3.75) | (5.00, 7.50) | (3.00, 11.50) |
| Range | (1.00, 50.00) | (2.00, 28.75) | (2.00, 8.33) | (2.00, 4.09) | (2.00, 8.00) | (1.00, 50.00) |
| MDE with metabolic acidosis |  |  |  |  |  |  |
| Number of patients with event during follow-up | 55 (28.8) | 14 (43.8) | 9 (28.1) | 4 (11.1) | 5 (20.8) | 28 (26.4) |
| Length of stay (days), per-patient-per-MDE |  |  |  |  |  |  |
| Mean±SD | 8.76±9.39 | 11.14±6.27 | 5.65±3.78 | 4.25±2.63 | 6.80±3.70 | 9.48±12.13 |
| Median | 5.00 | 13.57 | 5.00 | 3.50 | 7.00 | 4.00 |
| IQR | (3.00, 12.50) | (4.70, 15.75) | (3.00, 6.50) | (2.75, 5.00) | (5.00, 8.00) | (2.88, 11.63) |
| Range | (1.00, 50.00) | (3.00, 20.67) | (2.00, 14.00) | (2.00, 8.00) | (2.00, 12.00) | (1.00, 50.00) |
| MDE with hyperammonemia |  |  |  |  |  |  |
| Number of patients with event during follow-up | 26 (13.6) | 14 (43.8) | 5 (15.6) | 3 (8.3) | 2 (8.3) | 6 (5.7) |
| Length of stay (days), per-patient-per-MDE |  |  |  |  |  |  |
| Mean±SD | 10.50±10.80 | 15.10±14.01 | 3.68±1.95 | 3.07±0.13 | 5.50±3.54 | 8.25±5.25 |
| Median | 7.00 | 12.58 | 3.00 | 3.00 | 5.50 | 7.25 |
| IQR | (3.75, 13.64) | (4.63, 19.31) | (2.00, 5.05) | (3.00, 3.11) | (4.25, 6.75) | (4.38, 10.50) |
| Range | (2.00, 53.00) | (2.00, 53.00) | (2.00, 6.36) | (3.00, 3.22) | (3.00, 8.00) | (3.00, 17.00) |
| **Hospitalization with PA signs and symptoms** |  |  |  |  |  |  |
| Vomiting |  |  |  |  |  |  |
| Number of patients with hospitalization during follow-up | 53 (27.7) | 15 (46.9) | 11 (34.4) | 7 (19.4) | 5 (20.8) | 23 (21.7) |
| Length of stay (days), per-patient-per-MDE |  |  |  |  |  |  |
| Mean±SD | 6.37±7.06 | 8.01±6.12 | 6.88±9.78 | 3.20±0.94 | 5.09±1.82 | 5.47±6.81 |
| Median | 4 | 6.22 | 4 | 3.5 | 5 | 3 |
| IQR | (3.00, 6.67) | (3.65, 11.69) | (2.95, 5.31) | (2.50, 3.72) | (4.43, 5.00) | (3.00, 5.25) |
| Range | (1.00, 36.00) | (2.00, 23.50) | (2.00, 36.00) | (2.00, 4.50) | (3.00, 8.00) | (1.00, 34.00) |
| Seizures |  |  |  |  |  |  |
| Number of patients with hospitalization during follow-up | 33 (17.3) | 8 (25.0) | 9 (28.1) | 6 (16.7) | 2 (8.3) | 14 (13.2) |
| Length of stay (days), per-patient-per-MDE |  |  |  |  |  |  |
| Mean±SD | 8.37±6.25 | 11.76±9.06 | 5.46±2.46 | 4.49±2.57 | 6.00±1.41 | 8.56±5.82 |
| Median | 6.25 | 10.92 | 5 | 3.94 | 6 | 6.23 |
| IQR | (4.00, 10.00) | (3.88, 15.69) | (4.00, 6.96) | (3.65, 4.00) | (5.50, 6.50) | (5.00, 11.25) |
| Range | (2.00, 26.00) | (2.00, 26.00) | (2.00, 9.93) | (2.00, 9.50) | (5.00, 7.00) | (2.00, 21.00) |
| Anorexia/failure to feed |  |  |  |  |  |  |
| Number of patients with hospitalization during follow-up | 28 (14.7) | 12 (37.5) | 6 (18.8) | 2 (5.6) | 4 (16.7) | 10 (9.4) |
| Length of stay (days), per-patient-per-MDE |  |  |  |  |  |  |
| Mean±SD | 15.41±25.14 | 15.78±14.58 | 6.27±4.33 | 3.00±0.00 | 5.25±2.50 | 25.11±40.19 |
| Median | 6.03 | 9.17 | 5.63 | 3 | 5.5 | 5.46 |
| IQR | (4.25, 19.33) | (5.96, 25.00) | (3.75, 6.58) | (3.00, 3.00) | (4.25, 6.50) | (4.18, 26.50) |
| Range | (2.00, 131.00) | (3.00, 53.00) | (2.00, 14.33) | (3.00, 3.00) | (2.00, 8.00) | (2.00, 131.00) |

**Abbreviations:** IQR, interquartile range; MDE, metabolic decompensation event; PA, propionic acidemia; SD, standard deviation.

**Note:**

[1] MDE was defined as a diagnosis of metabolic acidosis or hyperammonemia occurring in the inpatient setting.

## Supplemental Table S2. Demographic and clinical characteristics of patients with PA who experienced any MDEs (in inpatient setting) and matched control subjects without PA

|  | **All patients with PA with MDE** | **Age stratum** | | | | | | | | | |
| --- | --- | --- | --- | --- | --- | --- | --- | --- | --- | --- | --- |
|  |  | **0–2 years** | | **3–6 years** | | **7–12 years** | | **13–17 years** | | **≥18 years** | |
|  |  |  |  |  |  |  |  |  |  |  |  |
|  |  | **PA with MDE** | **Non-PA controls** | **PA with MDE** | **Non-PA controls** | **PA with MDE** | **Non-PA controls** | **PA with MDE** | **Non-PA controls** | **PA with MDE** | **Non-PA controls** |
|  | **N=60** | **N=16** | **N=16** | **N=14** | **N=14** | **N=9** | **N=9** | **N=6** | **N=6** | **N=34** | **N=34** |
| **Demographic characteristics** |  |  |  |  |  |  |  |  |  |  |  |
| Age, years | 25.2±25.3 | 0.6±0.7 | 0.5±0.7 | 3.7±1.2 | 3.7±1.2 | 7.2±0.5 | 7.2±0.5 | 16.2±1.4 | 16.2±1.4 | 42.7±20.4 | 42.7±20.4 |
| Female | 26 (43.3) | 9 (56.3) | 9 (56.3) | 5 (35.7) | 5 (35.7) | 2 (22.2) | 2 (22.2) | 2 (33.3) | 2 (33.3) | 13 (38.2) | 13 (38.2) |
| Census region |  |  |  |  |  |  |  |  |  |  |  |
| Midwest | 23 (38.3) | 8 (50.0) | 8 (50.0) | 7 (50.0) | 7 (50.0) | 4 (44.4) | 4 (44.4) | 4 (66.7) | 4 (66.7) | 11 (32.4) | 11 (32.4) |
| South | 19 (31.7) | 5 (31.3) | 5 (31.3) | 4 (28.6) | 4 (28.6) | 3 (33.3) | 3 (33.3) | 0 (0.0) | 0 (0.0) | 11 (32.4) | 11 (32.4) |
| Northeast | 13 (21.7) | 3 (18.8) | 3 (18.8) | 3 (21.4) | 3 (21.4) | 2 (22.2) | 2 (22.2) | 2 (33.3) | 2 (33.3) | 7 (20.6) | 7 (20.6) |
| West | 5 (8.3) | 0 (0.0) | 0 (0.0) | 0 (0.0) | 0 (0.0) | 0 (0.0) | 0 (0.0) | 0 (0.0) | 0 (0.0) | 5 (14.7) | 5 (14.7) |
| Unknown | 0 (0.0) | 0 (0.0) | 0 (0.0) | 0 (0.0) | 0 (0.0) | 0 (0.0) | 0 (0.0) | 0 (0.0) | 0 (0.0) | 0 (0.0) | 0 (0.0) |
| Index year |  |  |  |  |  |  |  |  |  |  |  |
| 2015 | 11 (18.3) | 4 (25.0) | 4 (25.0) | 1 (7.1) | 1 (7.1) | 1 (11.1) | 1 (11.1) | 4 (66.7) | 4 (66.7) | 1 (2.9) | 1 (2.9) |
| 2016 | 12 (20.0) | 2 (12.5) | 2 (12.5) | 5 (35.7) | 5 (35.7) | 1 (11.1) | 1 (11.1) | 2 (33.3) | 2 (33.3) | 5 (14.7) | 5 (14.7) |
| 2017 | 5 (8.3) | 1 (6.3) | 1 (6.3) | 0 (0.0) | 0 (0.0) | 2 (22.2) | 2 (22.2) | 0 (0.0) | 0 (0.0) | 4 (11.8) | 3 (8.8) |
| 2018 | 12 (20.0) | 3 (18.8) | 3 (18.8) | 3 (21.4) | 3 (21.4) | 1 (11.1) | 1 (11.1) | 0 (0.0) | 0 (0.0) | 9 (26.5) | 10 (29.4) |
| 2019 | 7 (11.7) | 1 (6.3) | 1 (6.3) | 2 (14.3) | 2 (14.3) | 1 (11.1) | 1 (11.1) | 0 (0.0) | 0 (0.0) | 7 (20.6) | 7 (20.6) |
| 2020 | 8 (13.3) | 3 (18.8) | 3 (18.8) | 2 (14.3) | 2 (14.3) | 3 (33.3) | 3 (33.3) | 0 (0.0) | 0 (0.0) | 5 (14.7) | 5 (14.7) |
| 2021 | 5 (8.3) | 2 (12.5) | 2 (12.5) | 1 (7.1) | 1 (7.1) | 0 (0.0) | 0 (0.0) | 0 (0.0) | 0 (0.0) | 3 (8.8) | 3 (8.8) |
| Flu season (September–March)^1^ | 41 (68.3) | 13 (81.3) | 13 (81.3) | 7 (50.0) | 7 (50.0) | 1 (11.1) | 1 (11.1) | 6 (100.0) | 6 (100.0) | 18 (52.9) | 19 (55.9) |
| Insurance type |  |  |  |  |  |  |  |  |  |  |  |
| Commercial/self-insured | 51 (85.0) | 15 (93.8) | 15 (93.8) | 14 (100.0) | 14 (100.0) | 9 (100.0) | 9 (100.0) | 6 (100.0) | 6 (100.0) | 26 (76.5) | 26 (76.5) |
| Medicare | 7 (11.7) | 0 (0.0) | 1 (6.3) | 0 (0.0) | 0 (0.0) | 0 (0.0) | 0 (0.0) | 0 (0.0) | 0 (0.0) | 7 (20.6) | 1 (2.9) |
| Medicaid | 2 (3.3) | 1 (6.3) | 0 (0.0) | 0 (0.0) | 0 (0.0) | 0 (0.0) | 0 (0.0) | 0 (0.0) | 0 (0.0) | 1 (2.9) | 7 (20.6) |
| **Clinical characteristics** |  |  |  |  |  |  |  |  |  |  |  |
| PA symptoms | 52 (86.7) | **14 (87.5)** | **7 (43.8)** | **13 (92.9)** | **3 (21.4)** | 4 (44.4) | 3 (33.3) | 4 (66.7) | 0 (0.0) | **30 (88.2)** | **3 (8.8)** |
| Anorexia/failure to feed | 27 (45.0) | **13 (81.3)** | **5 (31.3)** | **10 (71.4)** | **2 (14.3)** | 3 (33.3) | 2 (22.2) | 4 (66.7) | 0 (0.0) | 5 (14.7) | 0 (0.0) |
| Vomiting | 26 (43.3) | **8 (50.0)** | **1 (6.3)** | **10 (71.4)** | **0 (0.0)** | 3 (33.3) | 0 (0.0) | 2 (33.3) | 0 (0.0) | **12 (35.3)** | **1 (2.9)** |
| Metabolic acidosis | 40 (66.7) | **12 (75.0)** | **0 (0.0)** | 4 (28.6) | 0 (0.0) | 1 (11.1) | 0 (0.0) | 3 (50.0) | 0 (0.0) | **24 (70.6)** | **0 (0.0)** |
| Seizures | 14 (23.3) | 4 (25.0) | 1 (6.3) | 3 (21.4) | 1 (7.1) | 4 (44.4) | 1 (11.1) | 2 (33.3) | 0 (0.0) | 9 (26.5) | 2 (5.9) |
| Hyperammonemia | 13 (21.7) | **9 (56.3)** | **0 (0.0)** | 4 (28.6) | 0 (0.0) | 2 (22.2) | 0 (0.0) | 1 (16.7) | 0 (0.0) | 1 (2.9) | 0 (0.0) |
| PA-related comorbidities |  |  |  |  |  |  |  |  |  |  |  |
| Metabolism-related conditions | 12 (20.0) | **6 (37.5)** | **0 (0.0)** | **8 (57.1)** | **0 (0.0)** | 1 (11.1) | 0 (0.0) | 1 (16.7) | 0 (0.0) | 2 (5.9) | 0 (0.0) |
| Other BCAA/FA metabolism disorders^2^ | 3 (5.0) | 1 (6.3) | 0 (0.0) | 4 (28.6) | 0 (0.0) | 1 (11.1) | 0 (0.0) | 0 (0.0) | 0 (0.0) | 0 (0.0) | 0 (0.0) |
| Methylmalonic acidemia | 4 (6.7) | 3 (18.8) | 0 (0.0) | 1 (7.1) | 0 (0.0) | 0 (0.0) | 0 (0.0) | 1 (16.7) | 0 (0.0) | 1 (2.9) | 0 (0.0) |
| Ketonuria | 5 (8.3) | 2 (12.5) | 0 (0.0) | 3 (21.4) | 0 (0.0) | 0 (0.0) | 0 (0.0) | 0 (0.0) | 0 (0.0) | 1 (2.9) | 0 (0.0) |
| Hypoglycemia | 1 (1.7) | 1 (6.3) | 0 (0.0) | 0 (0.0) | 0 (0.0) | 0 (0.0) | 0 (0.0) | 0 (0.0) | 0 (0.0) | 0 (0.0) | 0 (0.0) |
| Cytopenias | 23 (38.3) | **11 (68.8)** | **0 (0.0)** | 2 (14.3) | 0 (0.0) | 1 (11.1) | 0 (0.0) | 1 (16.7) | 0 (0.0) | **10 (29.4)** | **0 (0.0)** |
| Anemia | 19 (31.7) | **8 (50.0)** | **0 (0.0)** | 1 (7.1) | 0 (0.0) | 1 (11.1) | 0 (0.0) | 1 (16.7) | 0 (0.0) | **10 (29.4)** | **0 (0.0)** |
| Neutropenia | 5 (8.3) | 4 (25.0) | 0 (0.0) | 1 (7.1) | 0 (0.0) | 0 (0.0) | 0 (0.0) | 0 (0.0) | 0 (0.0) | 0 (0.0) | 0 (0.0) |
| Thrombocytopenia | 5 (8.3) | 4 (25.0) | 0 (0.0) | 0 (0.0) | 0 (0.0) | 0 (0.0) | 0 (0.0) | 1 (16.7) | 0 (0.0) | 0 (0.0) | 0 (0.0) |
| Growth complications | 15 (25.0) | 8 (50.0) | 2 (12.5) | 5 (35.7) | 0 (0.0) | 3 (33.3) | 0 (0.0) | 3 (50.0) | 0 (0.0) | 4 (11.8) | 0 (0.0) |
| Growth impairment | 12 (20.0) | 5 (31.3) | 1 (6.3) | 5 (35.7) | 0 (0.0) | 3 (33.3) | 0 (0.0) | 3 (50.0) | 0 (0.0) | 4 (11.8) | 0 (0.0) |
| Failure to thrive | 7 (11.7) | 5 (31.3) | 1 (6.3) | 1 (7.1) | 0 (0.0) | 0 (0.0) | 0 (0.0) | 0 (0.0) | 0 (0.0) | 1 (2.9) | 0 (0.0) |
| Cardiac system conditions | 12 (20.0) | 1 (6.3) | 0 (0.0) | 0 (0.0) | 0 (0.0) | 1 (11.1) | 0 (0.0) | 5 (83.3) | 0 (0.0) | **9 (26.5)** | **0 (0.0)** |
| Cardiomyopathy | 7 (11.7) | 0 (0.0) | 0 (0.0) | 0 (0.0) | 0 (0.0) | 0 (0.0) | 0 (0.0) | 3 (50.0) | 0 (0.0) | **6 (17.6)** | **0 (0.0)** |
| Long QT syndrome | 5 (8.3) | 1 (6.3) | 0 (0.0) | 0 (0.0) | 0 (0.0) | 1 (11.1) | 0 (0.0) | 2 (33.3) | 0 (0.0) | 3 (8.8) | 0 (0.0) |
| Neurologic and CNS/PNS conditions | 31 (51.7) | 6 (37.5) | 2 (12.5) | **10 (71.4)** | **2 (14.3)** | **6 (66.7)** | **2 (22.2)** | 4 (66.7) | 0 (0.0) | **19 (55.9)** | **2 (5.9)** |
| Movement disorders | 15 (25.0) | 4 (25.0) | 0 (0.0) | 4 (28.6) | 0 (0.0) | 0 (0.0) | 0 (0.0) | 1 (16.7) | 0 (0.0) | **11 (32.4)** | **1 (2.9)** |
| Delayed development of the speech | 6 (10.0) | 1 (6.3) | 1 (6.3) | 6 (42.9) | 1 (7.1) | 2 (22.2) | 1 (11.1) | 0 (0.0) | 0 (0.0) | 3 (8.8) | 0 (0.0) |
| Intellectual disability | 13 (21.7) | 3 (18.8) | 0 (0.0) | 4 (28.6) | 0 (0.0) | 2 (22.2) | 0 (0.0) | 3 (50.0) | 0 (0.0) | **7 (20.6)** | **0 (0.0)** |
| Psychosis | 12 (20.0) | 2 (12.5) | 0 (0.0) | 3 (21.4) | 0 (0.0) | 2 (22.2) | 0 (0.0) | 2 (33.3) | 0 (0.0) | **7 (20.6)** | **0 (0.0)** |
| Autism | 4 (6.7) | 0 (0.0) | 0 (0.0) | 1 (7.1) | 0 (0.0) | 1 (11.1) | 0 (0.0) | 1 (16.7) | 0 (0.0) | 3 (8.8) | 0 (0.0) |
| Impaired hearing ability | 3 (5.0) | 0 (0.0) | 0 (0.0) | 1 (7.1) | 1 (7.1) | 0 (0.0) | 1 (11.1) | 1 (16.7) | 0 (0.0) | 2 (5.9) | 0 (0.0) |
| Other comorbidities |  |  |  |  |  |  |  |  |  |  |  |
| Anxiety | 13 (21.7) | 0 (0.0) | 0 (0.0) | 0 (0.0) | 0 (0.0) | 0 (0.0) | 0 (0.0) | 1 (16.7) | 0 (0.0) | 13 (38.2) | 8 (23.5) |
| Hyperlipidemia | 14 (23.3) | 0 (0.0) | 0 (0.0) | 0 (0.0) | 0 (0.0) | 0 (0.0) | 0 (0.0) | 1 (16.7) | 0 (0.0) | 14 (41.2) | 7 (20.6) |
| Type II diabetes | 15 (25.0) | 0 (0.0) | 0 (0.0) | 0 (0.0) | 0 (0.0) | 0 (0.0) | 0 (0.0) | 0 (0.0) | 0 (0.0) | **15 (44.1)** | **1 (2.9)** |
| Obesity | 11 (18.3) | 2 (12.5) | 0 (0.0) | 2 (14.3) | 0 (0.0) | 1 (11.1) | 1 (11.1) | 0 (0.0) | 0 (0.0) | 8 (23.5) | 5 (14.7) |
| Epilepsy | 6 (10.0) | 2 (12.5) | 0 (0.0) | 2 (14.3) | 0 (0.0) | 3 (33.3) | 0 (0.0) | 0 (0.0) | 0 (0.0) | 6 (17.6) | 1 (2.9) |
| Depression | 12 (20.0) | 1 (6.3) | 0 (0.0) | 0 (0.0) | 0 (0.0) | 0 (0.0) | 0 (0.0) | 2 (33.3) | 1 (16.7) | 9 (26.5) | 4 (11.8) |
| Asthma | 8 (13.3) | 1 (6.3) | 0 (0.0) | 1 (7.1) | 0 (0.0) | 0 (0.0) | 0 (0.0) | 2 (33.3) | 0 (0.0) | 4 (11.8) | 1 (2.9) |
| Type I diabetes | 6 (10.0) | 0 (0.0) | 0 (0.0) | 0 (0.0) | 0 (0.0) | 0 (0.0) | 0 (0.0) | 0 (0.0) | 0 (0.0) | 6 (17.6) | 1 (2.9) |
| CKD^3^ | 8 (13.3) | 0 (0.0) | 0 (0.0) | 1 (7.1) | 0 (0.0) | 0 (0.0) | 0 (0.0) | 0 (0.0) | 0 (0.0) | **9 (26.5)** | **0 (0.0)** |
| Thyroid disease | 6 (10.0) | 0 (0.0) | 0 (0.0) | 0 (0.0) | 0 (0.0) | 0 (0.0) | 0 (0.0) | 2 (33.3) | 0 (0.0) | 4 (11.8) | 0 (0.0) |
| Arthritis | 4 (6.7) | 0 (0.0) | 0 (0.0) | 0 (0.0) | 0 (0.0) | 0 (0.0) | 0 (0.0) | 0 (0.0) | 0 (0.0) | 4 (11.8) | 3 (8.8) |
| ADHD | 3 (5.0) | 0 (0.0) | 0 (0.0) | 2 (14.3) | 0 (0.0) | 0 (0.0) | 0 (0.0) | 0 (0.0) | 0 (0.0) | 1 (2.9) | 0 (0.0) |
| CCI | 1.7±2.1 | 0.1±0.3 | 0.0±0.0 | 0.9±1.5 | 0.1±0.3 | 0.2±0.4 | 0.0±0.0 | 2.0±1.6 | 0.0±0.0 | **2.7±2.3** | **0.2±0.4** |
| Select conditions^4^ |  |  |  |  |  |  |  |  |  |  |  |
| Diabetes without chronic complication | 15 (25.0) | 0 (0.0) | 0 (0.0) | 0 (0.0) | 0 (0.0) | 0 (0.0) | 0 (0.0) | 0 (0.0) | 0 (0.0) | **15 (44.1)** | **2 (5.9)** |
| Chronic pulmonary disease | 12 (20.0) | 1 (6.3) | 0 (0.0) | 1 (7.1) | 1 (7.1) | 0 (0.0) | 0 (0.0) | 2 (33.3) | 0 (0.0) | 8 (23.5) | 4 (11.8) |
| Mild liver disease | 9 (15.0) | 1 (6.3) | 0 (0.0) | 4 (28.6) | 0 (0.0) | 1 (11.1) | 0 (0.0) | 2 (33.3) | 0 (0.0) | 5 (14.7) | 1 (2.9) |
| Renal disease | 14 (23.3) | 0 (0.0) | 0 (0.0) | 3 (21.4) | 0 (0.0) | 0 (0.0) | 0 (0.0) | 2 (33.3) | 0 (0.0) | **12 (35.3)** | **0 (0.0)** |
| Congestive heart failure | 12 (20.0) | 0 (0.0) | 0 (0.0) | 0 (0.0) | 0 (0.0) | 0 (0.0) | 0 (0.0) | 3 (50.0) | 0 (0.0) | **11 (32.4)** | **0 (0.0)** |
| Diabetes with chronic complication | 10 (16.7) | 0 (0.0) | 0 (0.0) | 0 (0.0) | 0 (0.0) | 0 (0.0) | 0 (0.0) | 0 (0.0) | 0 (0.0) | **10 (29.4)** | **0 (0.0)** |
| Cerebrovascular disease | 3 (5.0) | 0 (0.0) | 0 (0.0) | 0 (0.0) | 0 (0.0) | 0 (0.0) | 0 (0.0) | 0 (0.0) | 0 (0.0) | 4 (11.8) | 0 (0.0) |
| **Treatments** |  |  |  |  |  |  |  |  |  |  |  |
| Medication/procedure |  |  |  |  |  |  |  |  |  |  |  |
| L-carnitine | 25 (41.7) | **11 (68.8)** | **0 (0.0)** | **9 (64.3)** | **0 (0.0)** | **7 (77.8)** | **0 (0.0)** | **6 (100.0)** | **0 (0.0)** | **6 (17.6)** | **0 (0.0)** |
| Any antibiotic use | 25 (41.7) | **8 (50.0)** | **2 (12.5)** | 5 (35.7) | 3 (33.3) | 2 (22.2) | 1 (11.1) | 5 (83.3) | 1 (16.7) | 10 (29.4) | 4 (11.8) |
| Carglumic acid | 4 (6.7) | 3 (18.8) | 0 (0.0) | 1 (7.1) | 0 (0.0) | 0 (0.0) | 0 (0.0) | 1 (16.7) | 0 (0.0) | 1 (2.9) | 0 (0.0) |
| Nutritional supplementation | 21 (35.0) | **9 (56.3)** | **0 (0.0)** | **6 (42.9)** | **0 (0.0)** | 3 (33.3) | 0 (0.0) | 4 (66.7) | 0 (0.0) | **8 (23.5)** | **0 (0.0)** |
| G-tube/NG-tube | 20 (33.3) | **10 (62.5)** | **0 (0.0)** | 5 (35.7) | 0 (0.0) | 5 (55.6) | 0 (0.0) | 2 (33.3) | 0 (0.0) | 5 (14.7) | 0 (0.0) |
| Hemodialysis | 7 (11.7) | 1 (6.3) | 0 (0.0) | 2 (14.3) | 0 (0.0) | 0 (0.0) | 0 (0.0) | 0 (0.0) | 0 (0.0) | 5 (14.7) | 0 (0.0) |
| Peritoneal dialysis, hemofiltration, or other continuous renal replacement therapies | 3 (5.0) | 1 (6.3) | 0 (0.0) | 0 (0.0) | 0 (0.0) | 0 (0.0) | 0 (0.0) | 0 (0.0) | 0 (0.0) | 2 (5.9) | 0 (0.0) |

Data are presented as mean±standard deviation or n (%). Characteristics were summarized during the 6-month period after the index date. Values in boldface indicate a statistically significant difference between patients with PA and non-PA control subjects (p<0.05).

**Abbreviations:** ADHD, attention-deficit/hyperactivity disorder; BCAA, branched-chain amino acid; CCI, Charlson Comorbidity Index; CKD, chronic kidney disease; CNS, central nervous system; FA, fatty acid; G-tube, gastronomy tube; NG-tube, nasogastric tube; PA, propionic acidemia; PNS, peripheral nervous system.

**Notes:**

[1] Patients with PA and non-PA control subjects were matched on exposure to flu during flu season (index date between September and March) as this could impact rates of metabolic decompensation.

[2] Other disorders of BCAA and FA metabolism included all conditions listed under International Classification of Diseases, 10th Revision, Clinical Modification code E71.x excluding methylmalonic acidemia (E71.120) and PA (E71.121).

[3] CKD included stage 3–5 disease or ESRD.

[4] Conditions with prevalence >5% in any age stratum are reported.

## Supplemental Table S3. Demographic and clinical characteristics of patients with PA who experienced no MDEs (in inpatient setting) and matched control subjects without PA

|  | **All patients with PA with no MDE** | **Age stratum** | | | | | | | | | |
| --- | --- | --- | --- | --- | --- | --- | --- | --- | --- | --- | --- |
|  |  | **0–2 years** | | **3–6 years** | | **7–12 years** | | **13–17 years** | | **≥18 years** | |
|  |  |  |  |  |  |  |  |  |  |  |  |
|  |  | **PA with no MDE** | **Non-PA controls** | **PA with no MDE** | **Non-PA controls** | **PA with no MDE** | **Non-PA controls** | **PA with no MDE** | **Non-PA controls** | **PA with no MDE** | **Non-PA controls** |
|  | **N=131** | **N=16** | **N=16** | **N=18** | **N=18** | **N=27** | **N=27** | **N=18** | **N=18** | **N=72** | **N=72** |
| **Demographic characteristics** |  |  |  |  |  |  |  |  |  |  |  |
| Age, years | 24.7±20.7 | 0.4±0.5 | 0.4±0.6 | 4.2±1.1 | 4.2±1.1 | 9.0±1.9 | 9.0±1.9 | 14.3±1.6 | 14.3±1.6 | 39.7±16.2 | 39.7±16.2 |
| Female | 69 (52.7) | 10 (62.5) | 10 (62.5) | 10 (55.6) | 10 (55.6) | 8 (29.6) | 8 (29.6) | 6 (33.3) | 6 (33.3) | 42 (58.3) | 42 (58.3) |
| Census region |  |  |  |  |  |  |  |  |  |  |  |
| Midwest | 45 (34.4) | 6 (37.5) | 6 (37.5) | 8 (44.4) | 8 (44.4) | 9 (33.3) | 9 (33.3) | 6 (33.3) | 6 (33.3) | 25 (34.7) | 26 (36.1) |
| South | 44 (33.6) | 9 (56.3) | 9 (56.3) | 4 (22.2) | 4 (22.2) | 9 (33.3) | 9 (33.3) | 4 (22.2) | 4 (22.2) | 24 (33.3) | 24 (33.3) |
| Northeast | 24 (18.3) | 0 (0.0) | 0 (0.0) | 2 (11.1) | 2 (11.1) | 6 (22.2) | 6 (22.2) | 2 (11.1) | 2 (11.1) | 16 (22.2) | 16 (22.2) |
| West | 17 (13.0) | 1 (6.3) | 1 (6.3) | 4 (22.2) | 4 (22.2) | 3 (11.1) | 3 (11.1) | 6 (33.3) | 6 (33.3) | 6 (8.3) | 6 (8.3) |
| Unknown | 1 (0.8) | 0 (0.0) | 0 (0.0) | 0 (0.0) | 0 (0.0) | 0 (0.0) | 0 (0.0) | 0 (0.0) | 0 (0.0) | 1 (1.4) | 0 (0.0) |
| Index year |  |  |  |  |  |  |  |  |  |  |  |
| 2015 | 13 (9.9) | 0 (0.0) | 0 (0.0) | 6 (33.3) | 6 (33.3) | 2 (7.4) | 2 (7.4) | 1 (5.6) | 1 (5.6) | 4 (5.6) | 4 (5.6) |
| 2016 | 34 (26.0) | 5 (31.3) | 5 (31.3) | 3 (16.7) | 3 (16.7) | 12 (44.4) | 12 (44.4) | 4 (22.2) | 4 (22.2) | 12 (16.7) | 12 (16.7) |
| 2017 | 10 (7.6) | 4 (25.0) | 4 (25.0) | 1 (5.6) | 1 (5.6) | 2 (7.4) | 2 (7.4) | 0 (0.0) | 0 (0.0) | 5 (6.9) | 5 (6.9) |
| 2018 | 16 (12.2) | 1 (6.3) | 1 (6.3) | 0 (0.0) | 1 (5.6) | 5 (18.5) | 5 (18.5) | 1 (5.6) | 1 (5.6) | 14 (19.4) | 14 (19.4) |
| 2019 | 23 (17.6) | 3 (18.8) | 3 (18.8) | 3 (16.7) | 2 (11.1) | 2 (7.4) | 2 (7.4) | 5 (27.8) | 5 (27.8) | 13 (18.1) | 13 (18.1) |
| 2020 | 19 (14.5) | 1 (6.3) | 1 (6.3) | 3 (16.7) | 3 (16.7) | 2 (7.4) | 2 (7.4) | 2 (11.1) | 2 (11.1) | 14 (19.4) | 14 (19.4) |
| 2021 | 16 (12.2) | 2 (12.5) | 2 (12.5) | 2 (11.1) | 2 (11.1) | 2 (7.4) | 2 (7.4) | 5 (27.8) | 5 (27.8) | 10 (13.9) | 10 (13.9) |
| Flu season (September–March)^1^ | 81 (61.8) | 6 (37.5) | 6 (37.5) | 13 (72.2) | 13 (72.2) | 14 (51.9) | 14 (51.9) | 6 (33.3) | 6 (33.3) | 43 (59.7) | 44 (61.1) |
| Insurance type |  |  |  |  |  |  |  |  |  |  |  |
| Commercial/self-insured | 123 (93.9) | 14 (87.5) | 14 (87.5) | 18 (100.0) | 18 (100.0) | 26 (96.3) | 26 (96.3) | 18 (100.0) | 18 (100.0) | 67 (93.1) | 67 (93.1) |
| Medicare | 4 (3.1) | 0 (0.0) | 2 (12.5) | 0 (0.0) | 0 (0.0) | 0 (0.0) | 1 (3.7) | 0 (0.0) | 0 (0.0) | 4 (5.6) | 1 (1.4) |
| Medicaid | 4 (3.1) | 2 (12.5) | 0 (0.0) | 0 (0.0) | 0 (0.0) | 1 (3.7) | 0 (0.0) | 0 (0.0) | 0 (0.0) | 1 (1.4) | 4 (5.6) |
| **Clinical characteristics** |  |  |  |  |  |  |  |  |  |  |  |
| PA symptoms | 56 (42.7) | 8 (50.0) | 4 (25.0) | 8 (44.4) | 2 (11.1) | 8 (29.6) | 3 (11.1) | 6 (33.3) | 1 (5.6) | **32 (44.4)** | **5 (6.9)** |
| Anorexia/failure to feed | 26 (19.8) | 6 (37.5) | 3 (18.8) | 5 (27.8) | 2 (11.1) | 1 (3.7) | 2 (7.4) | 5 (27.8) | 1 (5.6) | **12 (16.7)** | **0 (0.0)** |
| Vomiting | 24 (18.3) | 3 (18.8) | 1 (6.3) | **6 (33.3)** | **0 (0.0)** | 2 (7.4) | 1 (3.7) | 0 (0.0) | 0 (0.0) | **15 (20.8)** | **3 (4.2)** |
| Metabolic acidosis | 10 (7.6) | 1 (6.3) | 0 (0.0) | 1 (5.6) | 0 (0.0) | 1 (3.7) | 0 (0.0) | 0 (0.0) | 0 (0.0) | **7 (9.7)** | **0 (0.0)** |
| Seizures | 14 (10.7) | 1 (6.3) | 0 (0.0) | 3 (16.7) | 0 (0.0) | 4 (14.8) | 0 (0.0) | 1 (5.6) | 0 (0.0) | 6 (8.3) | 2 (2.8) |
| Hyperammonemia | 1 (0.8) | 0 (0.0) | 0 (0.0) | 0 (0.0) | 0 (0.0) | 0 (0.0) | 0 (0.0) | 0 (0.0) | 0 (0.0) | 1 (1.4) | 0 (0.0) |
| PA-related comorbidities |  |  |  |  |  |  |  |  |  |  |  |
| Metabolism-related conditions | 18 (13.7) | 4 (25.0) | 0 (0.0) | **6 (33.3)** | **0 (0.0)** | 5 (18.5) | 0 (0.0) | 1 (5.6) | 0 (0.0) | 4 (5.6) | 0 (0.0) |
| Other BCAA/FA metabolism disorders^2^ | 10 (7.6) | 1 (6.3) | 0 (0.0) | 4 (22.2) | 0 (0.0) | 1 (3.7) | 0 (0.0) | 1 (5.6) | 0 (0.0) | 3 (4.2) | 0 (0.0) |
| Methylmalonic acidemia | 5 (3.8) | 1 (6.3) | 0 (0.0) | 3 (16.7) | 0 (0.0) | 2 (7.4) | 0 (0.0) | 0 (0.0) | 0 (0.0) | 1 (1.4) | 0 (0.0) |
| Ketonuria | 3 (2.3) | 0 (0.0) | 0 (0.0) | 0 (0.0) | 0 (0.0) | 2 (7.4) | 0 (0.0) | 0 (0.0) | 0 (0.0) | 1 (1.4) | 0 (0.0) |
| Hypoglycemia | 2 (1.5) | 2 (12.5) | 0 (0.0) | 0 (0.0) | 0 (0.0) | 0 (0.0) | 0 (0.0) | 0 (0.0) | 0 (0.0) | 0 (0.0) | 0 (0.0) |
| Cytopenias | 10 (7.6) | 2 (12.5) | 0 (0.0) | 1 (5.6) | 0 (0.0) | 0 (0.0) | 0 (0.0) | 0 (0.0) | 0 (0.0) | **7 (9.7)** | **0 (0.0)** |
| Anemia | 7 (5.3) | 2 (12.5) | 0 (0.0) | 0 (0.0) | 0 (0.0) | 0 (0.0) | 0 (0.0) | 0 (0.0) | 0 (0.0) | 5 (6.9) | 0 (0.0) |
| Neutropenia | 1 (0.8) | 0 (0.0) | 0 (0.0) | 1 (5.6) | 0 (0.0) | 0 (0.0) | 0 (0.0) | 0 (0.0) | 0 (0.0) | 0 (0.0) | 0 (0.0) |
| Thrombocytopenia | 2 (1.5) | 0 (0.0) | 0 (0.0) | 0 (0.0) | 0 (0.0) | 0 (0.0) | 0 (0.0) | 0 (0.0) | 0 (0.0) | 2 (2.8) | 0 (0.0) |
| Growth complications | 16 (12.2) | 6 (37.5) | 1 (6.3) | 3 (16.7) | 0 (0.0) | 5 (18.5) | 0 (0.0) | 1 (5.6) | 0 (0.0) | 4 (5.6) | 0 (0.0) |
| Growth impairment | 14 (10.7) | 5 (31.3) | 0 (0.0) | 2 (11.1) | 0 (0.0) | 3 (11.1) | 0 (0.0) | 1 (5.6) | 0 (0.0) | 4 (5.6) | 0 (0.0) |
| Failure to thrive | 2 (1.5) | 1 (6.3) | 1 (6.3) | 1 (5.6) | 0 (0.0) | 2 (7.4) | 0 (0.0) | 0 (0.0) | 0 (0.0) | 0 (0.0) | 0 (0.0) |
| Cardiac system conditions | 5 (3.8) | 0 (0.0) | 0 (0.0) | 1 (5.6) | 0 (0.0) | 2 (7.4) | 0 (0.0) | 1 (5.6) | 0 (0.0) | 3 (4.2) | 0 (0.0) |
| Cardiomyopathy | 4 (3.1) | 0 (0.0) | 0 (0.0) | 1 (5.6) | 0 (0.0) | 2 (7.4) | 0 (0.0) | 1 (5.6) | 0 (0.0) | 2 (2.8) | 0 (0.0) |
| Long QT syndrome | 1 (0.8) | 0 (0.0) | 0 (0.0) | 0 (0.0) | 0 (0.0) | 0 (0.0) | 0 (0.0) | 1 (5.6) | 0 (0.0) | 1 (1.4) | 0 (0.0) |
| Neurologic and CNS/PNS conditions | 33 (25.2) | 5 (31.3) | 2 (12.5) | **7 (38.9)** | **1 (5.6)** | **10 (37.0)** | **1 (3.7)** | 4 (22.2) | 2 (11.1) | **16 (22.2)** | **5 (6.9)** |
| Movement disorders | 17 (13.0) | 2 (12.5) | 0 (0.0) | 4 (22.2) | 1 (5.6) | 3 (11.1) | 0 (0.0) | 2 (11.1) | 1 (5.6) | 9 (12.5) | 3 (4.2) |
| Delayed development of the speech | 10 (7.6) | 1 (6.3) | 0 (0.0) | 3 (16.7) | 1 (5.6) | 5 (18.5) | 1 (3.7) | 3 (16.7) | 1 (5.6) | 5 (6.9) | 0 (0.0) |
| Intellectual disability | 11 (8.4) | 1 (6.3) | 0 (0.0) | 2 (11.1) | 0 (0.0) | 3 (11.1) | 0 (0.0) | 3 (16.7) | 0 (0.0) | 5 (6.9) | 1 (1.4) |
| Psychosis | 7 (5.3) | 0 (0.0) | 0 (0.0) | 1 (5.6) | 0 (0.0) | 3 (11.1) | 0 (0.0) | 2 (11.1) | 0 (0.0) | 4 (5.6) | 1 (1.4) |
| Autism | 6 (4.6) | 0 (0.0) | 0 (0.0) | 1 (5.6) | 0 (0.0) | 3 (11.1) | 0 (0.0) | 2 (11.1) | 0 (0.0) | 3 (4.2) | 0 (0.0) |
| Impaired hearing ability | 3 (2.3) | 2 (12.5) | 2 (12.5) | 0 (0.0) | 0 (0.0) | 0 (0.0) | 0 (0.0) | 0 (0.0) | 0 (0.0) | 1 (1.4) | 1 (1.4) |
| Other comorbidities |  |  |  |  |  |  |  |  |  |  |  |
| Anxiety | 22 (16.8) | 0 (0.0) | 0 (0.0) | 2 (11.1) | 0 (0.0) | 2 (7.4) | 1 (3.7) | 4 (22.2) | 0 (0.0) | 17 (23.6) | 8 (11.1) |
| Hyperlipidemia | 19 (14.5) | 0 (0.0) | 0 (0.0) | 0 (0.0) | 0 (0.0) | 0 (0.0) | 0 (0.0) | 0 (0.0) | 0 (0.0) | 19 (26.4) | 13 (18.1) |
| Type II diabetes | 27 (20.6) | 0 (0.0) | 0 (0.0) | 1 (5.6) | 0 (0.0) | 1 (3.7) | 0 (0.0) | 2 (11.1) | 0 (0.0) | **23 (31.9)** | **2 (2.8)** |
| Obesity | 12 (9.2) | 0 (0.0) | 0 (0.0) | 1 (5.6) | 0 (0.0) | 0 (0.0) | 2 (7.4) | 0 (0.0) | 0 (0.0) | 12 (16.7) | 6 (8.3) |
| Epilepsy | 10 (7.6) | 0 (0.0) | 0 (0.0) | 3 (16.7) | 0 (0.0) | 4 (14.8) | 0 (0.0) | 1 (5.6) | 0 (0.0) | 3 (4.2) | 1 (1.4) |
| Depression | 9 (6.9) | 0 (0.0) | 0 (0.0) | 1 (5.6) | 1 (5.6) | 0 (0.0) | 1 (3.7) | 0 (0.0) | 0 (0.0) | 8 (11.1) | 3 (4.2) |
| Asthma | 9 (6.9) | 1 (6.3) | 1 (6.3) | 0 (0.0) | 1 (5.6) | 1 (3.7) | 1 (3.7) | 1 (5.6) | 0 (0.0) | 6 (8.3) | 2 (2.8) |
| Type I diabetes | 19 (14.5) | 1 (6.3) | 0 (0.0) | 0 (0.0) | 0 (0.0) | 5 (18.5) | 0 (0.0) | **6 (33.3)** | **0 (0.0)** | **9 (12.5)** | **1 (1.4)** |
| CKD^3^ | 6 (4.6) | 1 (6.3) | 0 (0.0) | 0 (0.0) | 0 (0.0) | 0 (0.0) | 0 (0.0) | 0 (0.0) | 0 (0.0) | 5 (6.9) | 0 (0.0) |
| Thyroid disease | 8 (6.1) | 1 (6.3) | 0 (0.0) | 0 (0.0) | 0 (0.0) | 1 (3.7) | 0 (0.0) | 1 (5.6) | 0 (0.0) | 5 (6.9) | 2 (2.8) |
| Arthritis | 7 (5.3) | 0 (0.0) | 0 (0.0) | 0 (0.0) | 0 (0.0) | 0 (0.0) | 0 (0.0) | 0 (0.0) | 0 (0.0) | 7 (9.7) | 2 (2.8) |
| ADHD | 3 (2.3) | 0 (0.0) | 0 (0.0) | 0 (0.0) | 1 (5.6) | 0 (0.0) | 2 (7.4) | 1 (5.6) | 1 (5.6) | 3 (4.2) | 0 (0.0) |
| CCI | 0.8±1.2 | 0.3±0.7 | 0.1±0.3 | 0.3±0.6 | 0.1±0.2 | **0.4±0.6** | **0.0±0.2** | **0.6±0.6** | **0.0±0.0** | **1.1±1.5** | **0.2±0.5** |
| Select conditions^4^ |  |  |  |  |  |  |  |  |  |  |  |
| Diabetes without chronic complication | 30 (22.9) | 1 (6.3) | 0 (0.0) | 1 (5.6) | 0 (0.0) | 4 (14.8) | 0 (0.0) | **6 (33.3)** | **0 (0.0)** | **20 (27.8)** | **2 (2.8)** |
| Chronic pulmonary disease | 12 (9.2) | 1 (6.3) | 1 (6.3) | 0 (0.0) | 1 (5.6) | 1 (3.7) | 1 (3.7) | 1 (5.6) | 0 (0.0) | **9 (12.5)** | **2 (2.8)** |
| Mild liver disease | 12 (9.2) | 0 (0.0) | 0 (0.0) | 4 (22.2) | 0 (0.0) | 3 (11.1) | 0 (0.0) | 1 (5.6) | 0 (0.0) | 5 (6.9) | 1 (1.4) |
| Renal disease | 9 (6.9) | 1 (6.3) | 0 (0.0) | 0 (0.0) | 0 (0.0) | 0 (0.0) | 0 (0.0) | 0 (0.0) | 0 (0.0) | **8 (11.1)** | **0 (0.0)** |
| Congestive heart failure | 7 (5.3) | 0 (0.0) | 0 (0.0) | 1 (5.6) | 0 (0.0) | 2 (7.4) | 0 (0.0) | 1 (5.6) | 0 (0.0) | 5 (6.9) | 1 (1.4) |
| Diabetes with chronic complication | 6 (4.6) | 0 (0.0) | 0 (0.0) | 0 (0.0) | 0 (0.0) | 0 (0.0) | 0 (0.0) | 1 (5.6) | 0 (0.0) | 5 (6.9) | 2 (2.8) |
| Cerebrovascular disease | 7 (5.3) | 0 (0.0) | 0 (0.0) | 0 (0.0) | 0 (0.0) | 0 (0.0) | 0 (0.0) | 0 (0.0) | 0 (0.0) | 7 (9.7) | 2 (2.8) |
| **Treatments** |  |  |  |  |  |  |  |  |  |  |  |
| Medication/procedure |  |  |  |  |  |  |  |  |  |  |  |
| L-carnitine | 26 (19.8) | 3 (18.8) | 0 (0.0) | 5 (27.8) | 0 (0.0) | **7 (25.9)** | **0 (0.0)** | 3 (16.7) | 0 (0.0) | **13 (18.1)** | **0 (0.0)** |
| Any antibiotic use | 26 (19.8) | 4 (25.0) | 5 (31.3) | 4 (22.2) | 4 (22.2) | 3 (11.1) | 2 (7.4) | 2 (11.1) | 1 (5.6) | 16 (22.2) | 13 (18.1) |
| Carglumic acid | 0 (0.0) | 0 (0.0) | 0 (0.0) | 0 (0.0) | 0 (0.0) | 0 (0.0) | 0 (0.0) | 0 (0.0) | 0 (0.0) | 0 (0.0) | 0 (0.0) |
| Nutritional supplementation | 21 (16.0) | 3 (18.8) | 0 (0.0) | 2 (11.1) | 0 (0.0) | 4 (14.8) | 0 (0.0) | 2 (11.1) | 0 (0.0) | **12 (16.7)** | **0 (0.0)** |
| G-tube/NG-tube | 12 (9.2) | 3 (18.8) | 0 (0.0) | 2 (11.1) | 0 (0.0) | 2 (7.4) | 0 (0.0) | 2 (11.1) | 0 (0.0) | 5 (6.9) | 0 (0.0) |
| Hemodialysis | 3 (2.3) | 1 (6.3) | 0 (0.0) | 0 (0.0) | 0 (0.0) | 0 (0.0) | 0 (0.0) | 0 (0.0) | 0 (0.0) | 2 (2.8) | 0 (0.0) |
| Peritoneal dialysis, hemofiltration, or other continuous renal replacement therapies | 1 (0.8) | 1 (6.3) | 0 (0.0) | 0 (0.0) | 0 (0.0) | 0 (0.0) | 0 (0.0) | 0 (0.0) | 0 (0.0) | 0 (0.0) | 0 (0.0) |

Data are presented as mean±standard deviation or n (%). Characteristics were summarized during the 6-month period after the index date. Values in boldface indicate a statistically significant difference between patients with PA and non-PA control subjects (p<0.05).

**Abbreviations:** ADHD, attention-deficit/hyperactivity disorder; BCAA, branched-chain amino acid; CCI, Charlson Comorbidity Index; CKD, chronic kidney disease; CNS, central nervous system; FA, fatty acid; G-tube, gastronomy tube; NG-tube, nasogastric tube; PA, propionic acidemia; PNS, peripheral nervous system.

**Notes:**

[1] Patients with PA and non-PA control subjects were matched on exposure to flu during flu season (index date between September and March) as this could impact rates of metabolic decompensation.

[2] Other disorders of BCAA and FA metabolism included all conditions listed under International Classification of Diseases, 10th Revision, Clinical Modification code E71.x excluding methylmalonic acidemia (E71.120) and PA (E71.121).

[3] CKD included stage 3–5 disease or ESRD.

[4] Conditions with prevalence >5% in any age stratum are reported.

## Supplemental Figure S1. Sensitivity analysis of rates of MDEs and hospitalization with PA signs and symptoms, defined as events occurring in inpatient or emergency room >24 hr setting, in patients with PA.

1. MDEs in inpatient or emergency room >24 hr setting


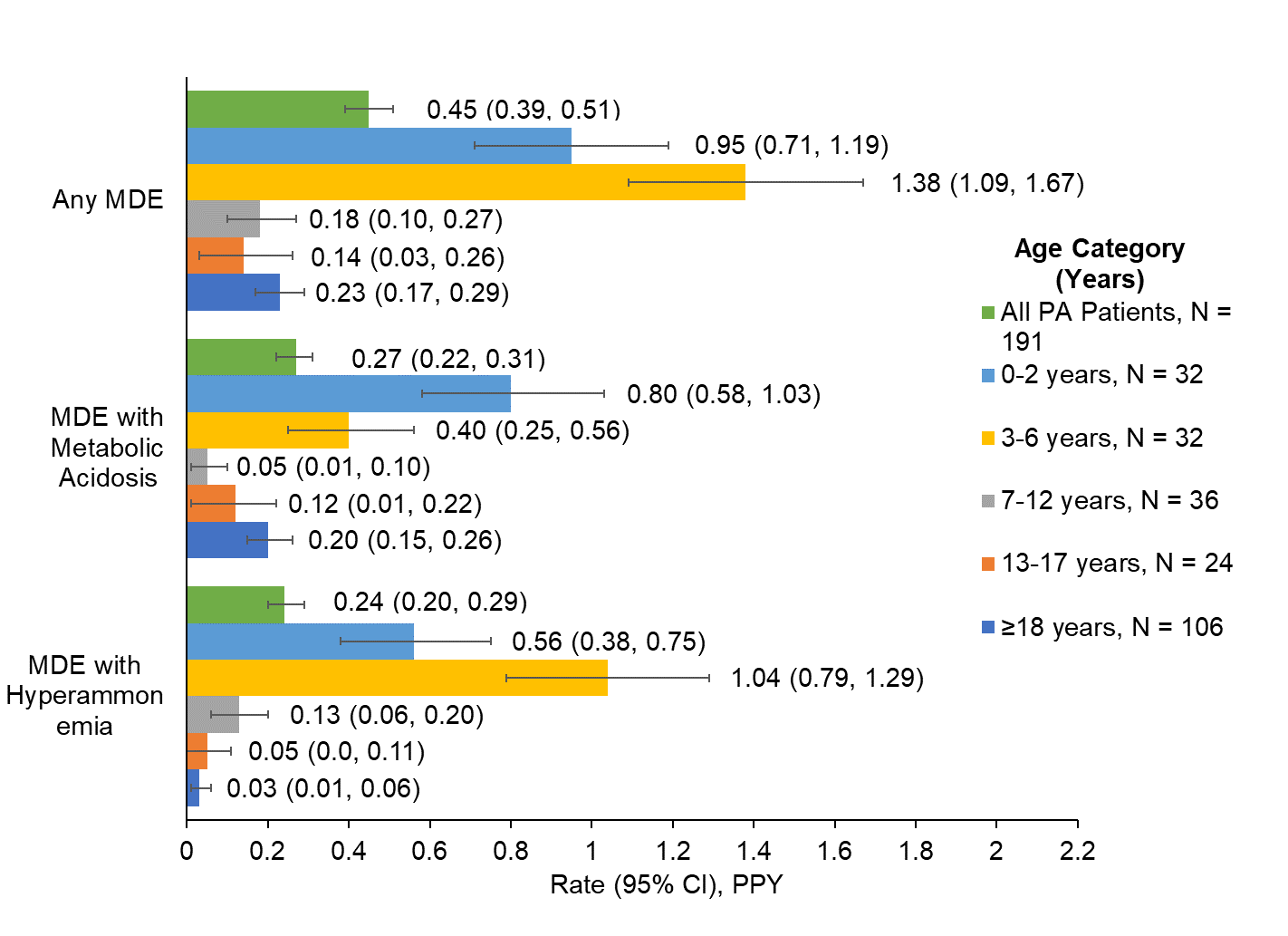


1. Hospitalizations or emergency room visits (>24 hr) with PA signs and symptoms


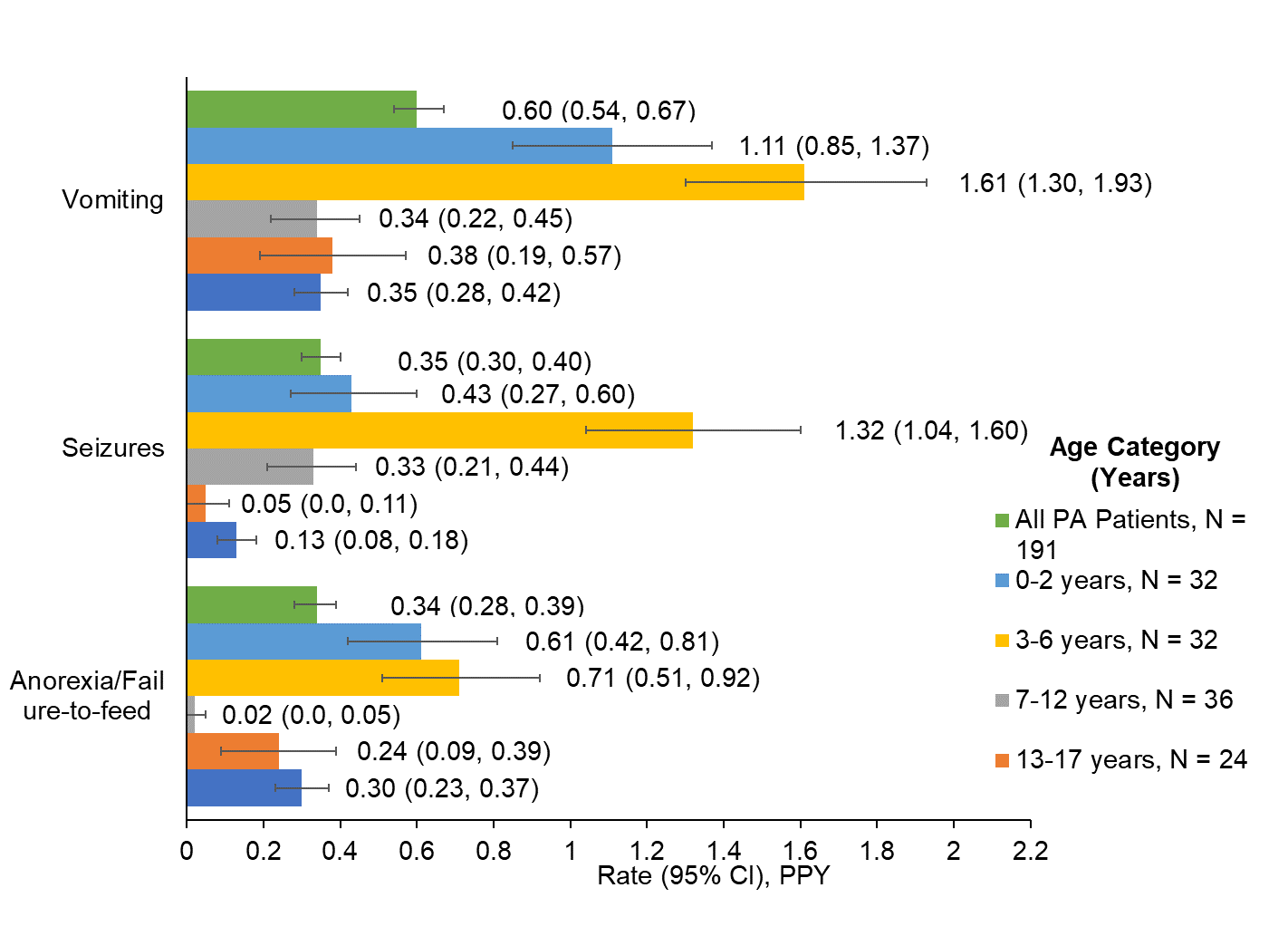


**Abbreviations:** CI, confidence interval; MDE, metabolic decompensation event; PA, propionic acidemia; PPY, per person-year.

## Supplemental Figure S2. Rates of MDEs^1^ and hospitalizations with PA signs and symptoms in patients with PA before and after the start of the COVID-19 pandemic (March 2020).

1. MDE

**
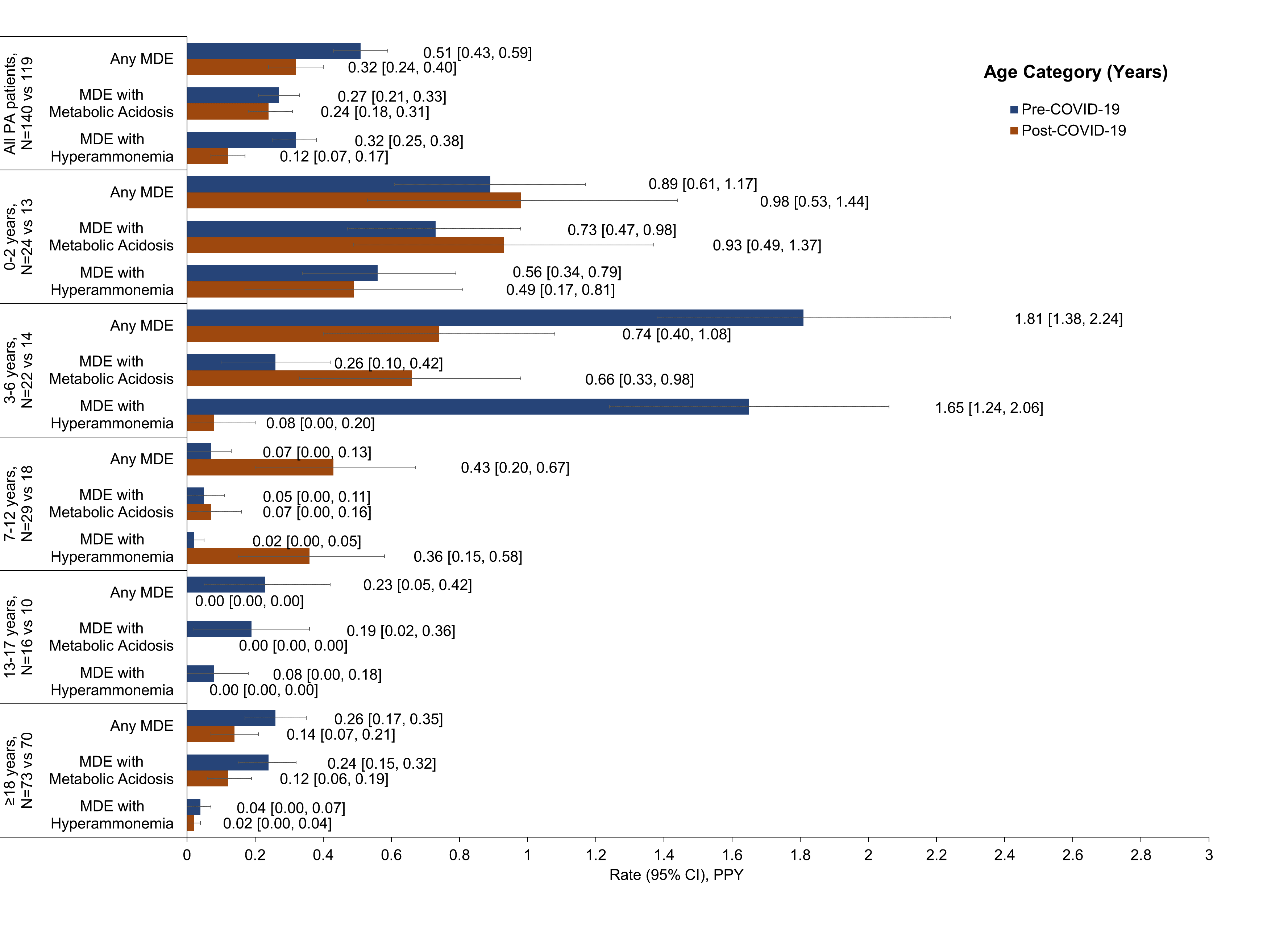
**

1. PA signs and symptoms


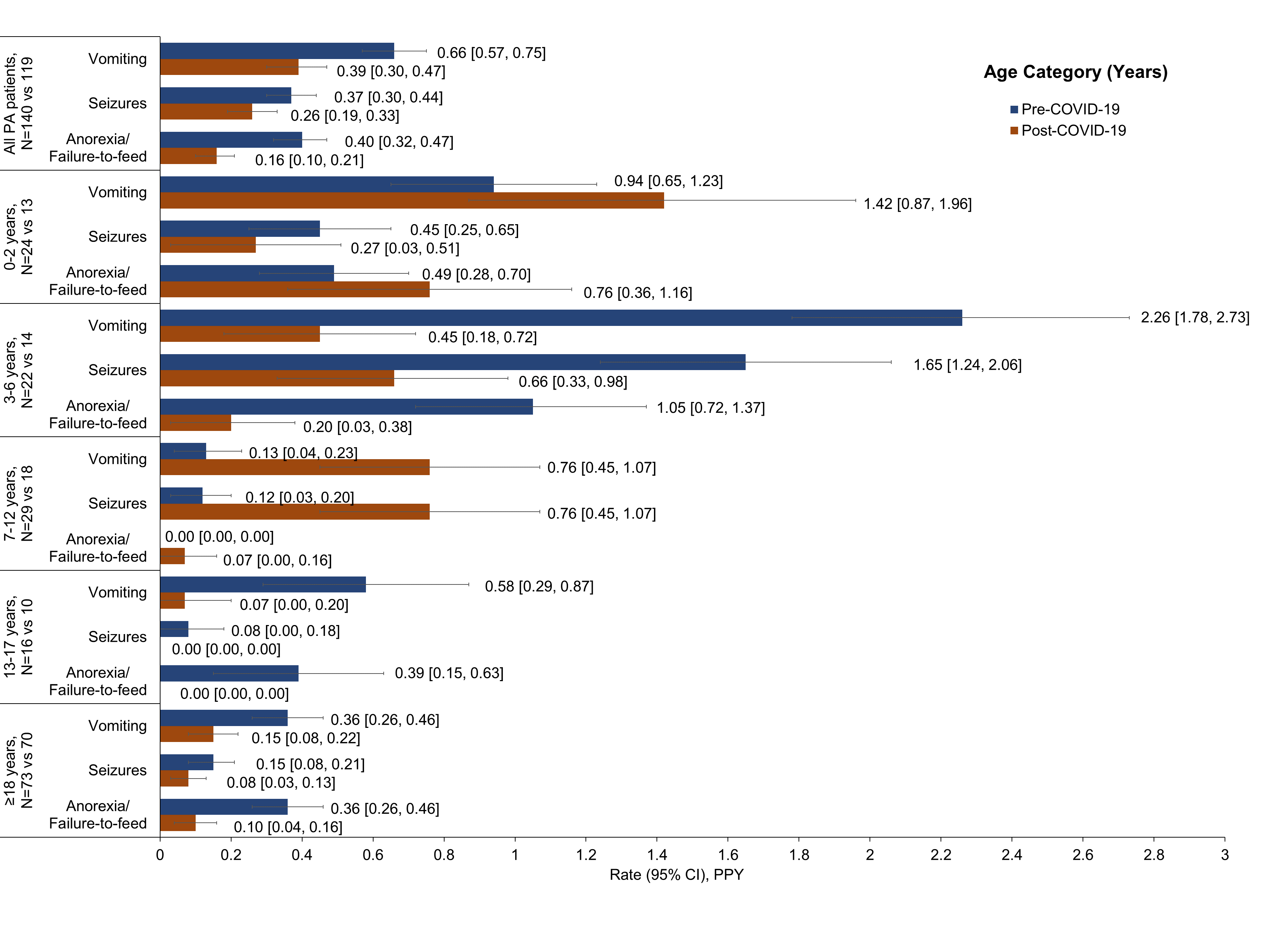


**Abbreviations:** CI, confidence interval; COVID-19, coronavirus disease 2019; MDE, metabolic decompensation event; PA, propionic acidemia; PPY, per person-year.

**Note:**

[1] MDE was defined as a diagnosis of metabolic acidosis or hyperammonemia occurring in the inpatient setting.
